# Investigating the impact of the English health inequalities strategy: time trend analysis

**DOI:** 10.1136/bmj.j3310

**Published:** 2017-07-26

**Authors:** Ben Barr, James Higgerson, Margaret Whitehead

**Affiliations:** Department of Public Health and Policy, Institute of Psychology, Health and Society, University of Liverpool, Liverpool L69 3GB, UK

## Abstract

**Objective** To investigate whether the English health inequalities strategy was associated with a decline in geographical health inequalities, compared with trends before and after the strategy.

**Design** Time trend analysis.

**Setting** Two groups of lower tier local authorities in England. The most deprived, bottom fifth and the rest of England.

**Intervention** The English health inequalities strategy—a cross government strategy implemented between 1997 and 2010 to reduce health inequalities in England. Trends in geographical health inequalities were assessed before (1983-2003), during (2004-12), and after (2013-15) the strategy using segmented linear regression.

**Main outcome measure** Geographical health inequalities measured as the relative and absolute differences in male and female life expectancy at birth between the most deprived local authorities in England and the rest of the country.

**Results** Before the strategy the gap in male and female life expectancy between the most deprived local authorities in England and the rest of the country increased at a rate of 0.57 months each year (95% confidence interval 0.40 to 0.74 months) and 0.30 months each year (0.12 to 0.48 months). During the strategy period this trend reversed and the gap in life expectancy for men reduced by 0.91 months each year (0.54 to 1.27 months) and for women by 0.50 months each year (0.15 to 0.86 months). Since the end of the strategy period the inequality gap has increased again at a rate of 0.68 months each year (−0.20 to 1.56 months) for men and 0.31 months each year (−0.26 to 0.88) for women. By 2012 the gap in male life expectancy was 1.2 years smaller (95% confidence interval 0.8 to 1.5 years smaller) and the gap in female life expectancy was 0.6 years smaller (0.3 to 1.0 years smaller) than it would have been if the trends in inequalities before the strategy had continued.

**Conclusion** The English health inequalities strategy was associated with a decline in geographical inequalities in life expectancy, reversing a previously increasing trend. Since the strategy ended, inequalities have started to increase again. The strategy may have reduced geographical health inequalities in life expectancy, and future approaches should learn from this experience. The concerns are that current policies are reversing the achievements of the strategy.

## Introduction

Between 1997 and 2010 the UK government implemented a comprehensive programme to reduce health inequalities in England,^1^ one of the most ambitious strategies of its kind.^2^ The strategy specifically focused on reducing geographical inequalities in life expectancy; with a target set to reduce by at least 10% the gap in life expectancy between the fifth of local authorities with the worst health and deprivation indicators (the Spearhead areas) and the population as a whole.^3^

The strategy focused on four themes^4^ ^5^: supporting families; engaging communities in tackling deprivation; improving prevention, treatment, and care; and tackling the underlying social determinants of health. Several government departments made 82 commitments across these four themes (see supplementary appendix 1).^6^ During the initial stages of the strategy, up to 2006, there was a broad focus across these four themes. By 2007 most of the departmental commitments had been met, at an estimated cost of more than £20bn ($26bn; €23bn) (see supplementary appendix 1).^2^ ^7^ Many actions were targeted at areas with high levels of socioeconomic deprivation, including several area based regeneration and health initiatives, and Sure Start children’s centres that provided early years child care and education.^5^ A new policy was introduced to allocate an increasing proportion of UK National Health Service resources to more deprived areas.^8^ Other actions targeted disadvantaged individuals and families, such as the introduction of the national minimum wage, tax and benefit changes to reduce child poverty, and interventions to improve education, housing, and employment.^5^ Actions that were focused on the health service included interventions to improve chronic disease management and access to primary care and smoking cessation services.^2^ Overall, this period in England was characterised by a large increase in public spending on social programmes and a focus across governments on widening opportunities for more disadvantaged areas, individuals, and families.^5^ ^9^ From 2006 greater emphasis was placed on the Spearhead areas. This included the setting of local targets for inequalities, aligned to national targets that local public sector organisations were obliged to report against,^3^ ^10^ and the establishment of a Health Inequalities National Support Team that provided technical advice for Spearhead areas to implement evidenced based approaches to reduce health inequalities^3^ ^10^ (see supplementary appendix 1 for a timeline for the main elements of the strategy).

The strategy came to an end with the change in government in 2010. While inequalities in some determinants of health had improved, including unemployment, child and pensioner poverty, housing quality, and educational attainment,^2^ ^11^ ^12^ ^13^ others remained stable or widened, including income inequality, smoking, and obesity.^2^ ^11^ ^14^ ^15^ The Department of Health’s own assessment in 2010, using data up to 2008 (the latest available at the time) estimated that the gap in life expectancy between the Spearhead areas and the country as whole had widened, and several commentators therefore concluded that the strategy had not been successful.^2^ ^3^ ^16^ ^17^ The effects of the strategy, however, may not have been fully realised by this time and this assessment did not consider any change from the pre-existing trend in health inequalities before the implementation of the strategy. Also, after the 2011 census, life expectancy estimates were revised based on new population estimates, and definitive data for the full strategy period only became available in 2013.^18^ More recently several studies have reported that inequalities in mortality did actually narrow between areas during the strategy period, based on their level of socioeconomic deprivation,^19^ ^20^ ^21^ and the Office for National Statistics (ONS) has reported that inequalities in male life expectancy between occupational socioeconomic groups also narrowed during this time.^22^ A recent study, however, found no evidence that the strategy had had an impact on educational inequalities in self assessed health, smoking, and obesity.^23^

It therefore remains unclear whether the English health inequalities strategy did or did not have an impact on geographical health inequalities. We investigated whether the period of the health inequalities strategy was associated with a reduction in the difference in life expectancy between the most disadvantaged local authorities and the country as a whole compared with trends before and after the strategy.

## Methods

### Setting and data sources

We used data from the UK Data Archive^24^ and the ONS on the annual number of deaths and population estimates for five year age groups for men and women in local authority areas across England between 1983 and 2015. Although the earliest data available were from 1979, we used data from 1983 because there are known issues with the quality of mortality data in 1981 and 1982 due to the registrars strike in those years.^25^ All data were mapped to 324 local authorities based on 2009 boundaries, excluding the City of London and Isles of Scilly because of their small population sizes. We used the income domain of the 2004 indices of multiple deprivation to identify the most deprived local authorities that included approximately 20% of the population of England (population weighted quintile). The income domain of the indices of multiple deprivation 2004 is a non-overlapping count of the numbers of people in each local authority on a low income and in receipt of means tested benefits or tax credits, or both.^26^ Supplementary appendix 11 gives the summary data and the geographical location of these most deprived local authorities.

### Analyses

Initially we calculated life expectancy^27^ at birth for men and women in the most deprived group of local authorities and the rest of England from 1983 to 2015 and the relative and absolute differences between these groups to investigate trends in inequalities before, during, and after the strategy was implemented. We then tested whether there was a statistically significant change in inequalities between the deprived group of local authorities and the rest of the country during the strategy period, compared with the period before and after. We calculated male and female life expectancy for each local authority area from 1983 to 2015. We then used this panel of data to estimate segmented regression models for male and female life expectancy, including linear spline terms for time with two breakpoints at the beginning and the end of the strategy period, and an interaction term between these time trend terms and a dummy variable indicating the most deprived group of local authorities (see supplementary appendix 2 for full model formula). All models included controls for local trends in unemployment, using annual data on the percentage of 16-64 year olds claiming unemployment benefits.^28^ This segmented regression model provided an estimation of the trend in the absolute difference in life expectancy between the most deprived local authorities and the rest of England during each period and whether there was a statistically significant change in this trend between periods. All models were weighted for the local authority population and included local authority fixed effects and clustered standard errors to adjust for the clustering of variance within local authorities (see supplementary appendix 2).

As the strategy developed incrementally (see supplementary appendix 1), and it is likely that there was a lag between implementation and any impacts on life expectancy, it was not possible to determine a priori at which time points we might expect the trend in inequalities to change. We therefore investigated empirically whether there was a statistically significant change in the trend in health inequalities around the beginning of the strategy period (1997 to 2006) and around the end of the strategy period (2008 to 2015). We used an iterative search procedure to identify which combination of breakpoints at the beginning and end of the strategy provided the best fit for the data by comparing all models with these alternative breakpoints^29^ (see supplementary appendix 4). In all further analyses we then used the model with breakpoints that best fitted the data.

Although the national target for the strategy was based on the gap in life expectancy between Spearhead areas and the rest of the country, we investigated the gap in life expectancy between the most income deprived local authorities and the rest of the country. The Spearhead areas were mainly selected because they had the worst health indicators in the country in 1995-97 rather than necessarily the worst levels of socioeconomic deprivation (see supplementary appendix 5). Health inequalities, however, are usually categorised as differences in health between groups defined by their socioeconomic status (eg, income) rather than their baseline health status.^30^ ^31^ ^32^ We also found that there was a high chance that the “Spearhead gap” would not narrow even if socioeconomic inequalities in health did narrow, and that there was also a high risk of biased conclusions from using this comparison owing to regression to the mean (see supplementary appendix 5). Most of the actions in the strategy were actually targeted generally at socioeconomically disadvantaged areas and groups rather than specifically at the Spearhead group, and therefore differences in life expectancy between the most income deprived local authorities and the rest of the country provide a measure of geographical health inequalities that was likely to be sensitive to the impact of the strategy. As a sensitivity analysis, we replicated the models using Spearhead and non-Spearhead areas rather than deprived areas and the rest of the country, and also investigated whether there was any additional increase in life expectancy in Spearhead areas compared with non-Spearhead areas after 2005, while adjusting for differential trends in deprived and non-deprived areas (see supplementary appendix 6).

### Robustness tests

To assess the robustness of our findings we subjected our analysis to several tests. We estimated models using the log of life expectancy in each local authority, including random rather than fixed effects, additionally including a random slope term, removing outliers, removing controls for the local unemployment rate, and using Driscoll and Kraay standard errors that are robust to serial autocorrelation.^33^ ^34^ We estimated models including a continuous term for deprivation rather than two groups of local authorities (see supplementary appendix 4). We investigated any non-linear relation between deprivation and change in life expectancy before, during, and after the strategy (see supplementary appendix 14). We estimated models adjusting for local internal and international migration rates (see supplementary appendix 4). To investigate differences in effect by age group we estimated models using age specific mortality rates (see supplementary appendix 12). To identify whether trends in the gap in life expectancy between groups of local authorities reflected changes in inequalities across neighbourhoods within these groups of local authorities, we provided additional analyses (see supplementary appendix 8) investigating the change in inequalities between small neighbourhoods within the most deprived group of local authorities and within the rest of the country.

### Patient involvement

No patients were involved in setting the research question or the outcome measures, nor were they involved in developing plans for design or implementation of the study. No patients were asked to advise on interpretation or writing up of results. There are no plans to disseminate the results of the research to study participants or the relevant patient community.

## Results

Figure 1 shows the trend in life expectancy in the most deprived local authorities and the rest of England between 1983 and 2015, and the relative and absolute differences in life expectancy between these two groups of local authorities. Life expectancy generally increased over this period for all groups; however, between 2012 and 2015 life expectancy declined slightly, particularly in the most deprived local authorities (fig 1). The gap in male and female life expectancy between the most deprived local authorities and the rest of England increased in the period before the introduction of the English health inequalities strategy, up to around 2000 to 2003, and then declined during the strategy period and increased again between 2012 and 2015.

**Figure f1:**
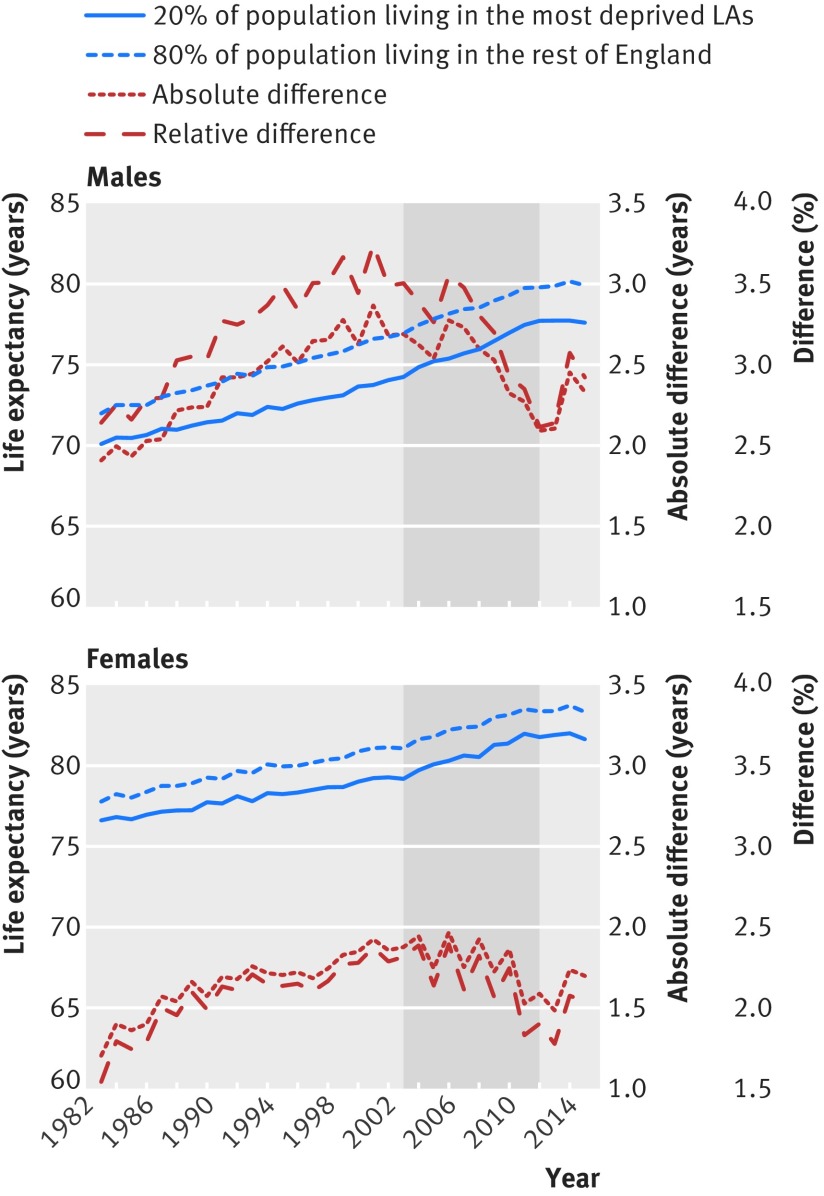
**Fig 1** Trends in life expectancy in the most deprived local authorities and the rest of England, and the relative and absolute differences 1983-2015

Comparing segmented regression models with different breakpoints indicated that breakpoints at 2003 and 2012 provided the best fit for the data (see supplementary appendix 7). Table 1 shows the trend in health inequalities estimated from the segmented regression models during the three periods defined by these breakpoints—before (1983-2003), during (2004-12), and after (2013-15) the health inequalities strategy.

**Table 1 tbl1:** Trend in absolute inequalities in life expectancy between the most deprived local authorities and the rest of England, before, during, and after the health inequalities strategy. Trend is shown as the annual increase or decrease (minus values) in the absolute gap in life expectancy (months)

Period, by sex	Annual change (months) in absolute gap in life expectancy between most deprived 20% of LAs and rest of England (95% CI)	P value for trend	P value for change in trend from previous period
Men:			
Before (1983-2003)	0.57 (0.40 to 0.74)	<0.001	
During (2004-12)	−0.91 (−1.27 to −0.54)	<0.001	<0.001
After (2013-15)	0.68 (−0.20 to 1.56)	0.13	<0.001
n=10 692 LA years, R^2^=0.74			
Women:			
Before (1983-2003)	0.3 (0.12 to 0.48)	<0.001	
During (2004-12)	−0.5 (−0.86 to −0.15)	0.01	<0.001
After (2013-15)	0.31 (−0.26 to 0.88)	0.29	0.01
n=10 692 LA years, R^2^=0.65			

LA=local authority.

Estimates based on fixed effects regression model using LA panel dataset of life expectancy from 1983 to 2015, also adjusted for local unemployment rates.

Before the strategy, the gap in male and female life expectancy between the most deprived local authorities in England and the rest of the country increased at a rate of 0.57 months each year (95% confidence interval 0.40 to 0.74 months) and 0.30 months each year (0.12 to 0.48 months), respectively. During the strategy period this trend reversed and the gap in life expectancy reduced by 0.91 months each year (0.54 to 1.27 months) for men and by 0.50 months each year (0.15 to 0.86 months) for women. Since the end of the strategy period the inequality gap has increased again at rate of 0.68 months each year (−0.20 to 1.56 months) for men and 0.31 months each year (−0.26 to 0.88 months) for women. For both male and female life expectancy, there was a statistically significant change in the trend in inequalities before and after the strategy (P<0.001). By 2012 the gap in male life expectancy was 1.2 years (34%) smaller (95% confidence interval 0.8 to 1.5 years smaller) and the gap in female life expectancy was 0.6 years (28%) smaller (0.3 to 1.0 years smaller) than it would have been if the trends in inequalities before the implementation of strategy had continued.

Additional analysis indicated that the reduction in inequalities during the strategy period was largely due to decreased inequalities in mortality in those aged less than 65 years (see supplementary appendix 12). The reductions in inequalities during the strategy period were also particularly due to greater improvements in the most deprived areas, rather than proportional improvements across all levels of deprivation (see supplementary appendix 14). We also found that there was a greater decline in inequalities between small neighbourhoods within the deprived group of local authorities during the strategy period than within the rest of the country (see supplementary appendix 8), suggesting that the decline in inequalities observed at the local authority level was achieved in part through reducing inequalities within the deprived local authorities.

Our results were similar when using alternative model specifications and when removing potential outliers (see supplementary appendix 4). We found that there was a statistically significant upturn in the trend in life expectancy in both deprived areas and the rest of the country in 2003; however, this change in trend was greater in the more deprived areas, hence inequalities narrowed. Similarly, the downturn in life expectancy from 2012 affected both deprived areas and the rest of the country, but this change in trend was greatest in more deprived areas, widening inequalities (see supplementary appendices 3 and 6). We found that the narrowing of the gap in life expectancy between Spearhead areas and the rest of the country did not occur until after 2005 and was less pronounced for women than men. The national target to reduce the gap between Spearhead areas and England as a whole by at least 10% was achieved for male life expectancy but not for female life expectancy. We found that male and female life expectancy increased after 2005 in Spearhead areas compared with non-Spearhead areas by an additional 2.8 months (95% confidence interval 0.02 to 5.5 months) and 3.14 months (0.97 to 5.31 months), respectively, after adjusting for the differential trends in deprived and non-deprived areas. In other words, there appeared to be an additional Spearhead effect narrowing inequalities after 2005 (see supplementary appendix 6).

## Discussion 

We found that absolute and relative inequalities in life expectancy between the most deprived English local authorities and the rest of the country, increased before the English health inequalities strategy, declined during the strategy period, and have increased since the strategy came to an end. This study provides the first evidence indicating that the English health inequalities strategy may have reduced geographical health inequalities in life expectancy and raises concerns that current policies are reversing these gains.

### Comparison with previous research

Our conclusions differ from previous, less favourable assessments of the impact of the strategy.^2^ ^3^ ^16^ ^17^ ^23^ Most of these assessments were based on the Department of Health’s own estimate that the gap in life expectancy between Spearhead areas and the country as a whole had not narrowed.^17^ There are several reasons for this difference in conclusions. Firstly, while the gap in life expectancy between income deprived areas and the rest of the country narrowed from 2003, this was not reflected in a narrowing of the Spearhead gap until 2006. This is probably because most of the actions of the strategy before 2006 were targeted generally at socioeconomically deprived areas and groups and not specifically at Spearhead areas. We do, however, observe a narrowing of the Spearhead gap from 2006. Secondly, in contrast with the Department of Health’s assessment we compared trends in inequalities during the strategy period with pre-existing trends, using life expectancy estimates revised after the 2011 census. Both of these factors led to a clearer indication that the strategy was associated with a narrowing of inequalities (see supplementary appendix 10). Other more recent analysis is consistent with our findings, also reporting a narrowing of inequalities in life expectancy during the period of the health inequalities strategy.^22^ ^35^ A recent study, however, found no evidence that the strategy had had an impact in inequalities in self reported health, smoking, and obesity between educational groups.^23^ Other studies have also reported widening inequalities in self reported health during the strategy period that were largely driven by increasing inequalities in mental health.^36^ ^37^ Alongside our findings this suggests that while inequalities in life expectancy decreased during the period of the strategy, inequalities in mental health may have increased. Others have also reported that inequalities in smoking remained fairly stable during the strategy period,^14^ while inequalities in obesity increased,^15^ suggesting that the narrowing of inequalities in life expectancy we observed was not due to reduced inequalities in these lifestyle factors.

### Role of economic trends, public investment, and welfare policy during strategy period

We found that there was a reversal in the trend in health inequalities from 2003. We cannot conclusively say whether this change would or would not have happened in the absence of the strategy. During the 1980s and 1990s there were relatively high levels of unemployment and increases in income inequality and poverty, whereas from the late 1990s to 2008 there was considerable economic stability, relatively low unemployment, and reductions in child and pensioner poverty (see supplementary appendix 9). These economic trends could in part explain changes in these health inequalities. Although some of these economic changes were due to global forces, some national policies such as the introduction of tax and pension credits, that were part of the health inequalities strategy, contributed to these economic trends by reducing levels of poverty.^5^ ^13^ From 2000 to 2010 there was also a noticeable upturn in public expenditure—in particular on health, education, housing, and local government (see supplementary appendix 9). This increased social investment may have led to the reduced health inequalities we observed. These investments, however, can also be seen as part of the health inequalities strategy. For example, increased investment in housing was necessary to achieve one of the departmental commitments of the strategy, to improve the quality of 370 000 homes.^7^ An important component of the health inequalities strategy was to influence the distribution of this increased investment, with NHS and local government expenditure growing most in the most deprived areas during the strategy period (see supplementary appendix 9). There was not always a clear distinction between policies that were part of the health inequalities strategy and policies that would have happened anyway in the absence of the strategy. Our analysis shows, however, that this period of increased social investment across the whole of government, targeted at disadvantaged areas and groups, was associated with a decline in health inequalities.

### Role of health service

From our analysis we were not able to determine the relative contribution of health service focused actions compared with those tackling the social determinants of health. We know there was a shift in NHS resources to more disadvantaged areas, leading to a substantial decline in inequalities in the provision and quality of primary care^21^ ^38^ ^39^ and a narrowing in inequalities in mortality amenable to healthcare.^19^ However, regeneration initiatives, improved child care, antipoverty measures, and improvements in housing may also have been important. The actions in the Spearhead areas, which started from 2006, focused on increasing uptake of smoking cessation services and drugs to control blood pressure and reduce cholesterol levels.^3^ We found that there was an additional decline in inequality, from 2006, that particularly affected the Spearhead areas, suggesting that these actions might have had an impact. Other studies also have found that some indicators of primary care quality disproportionately improved in Spearhead areas, even when accounting for a narrowing of inequalities between socioeconomically deprived and less deprived areas.^38^ ^39^

### Role of financial crisis and austerity policies

The reversal of the trend in health inequalities from 2012 could be related to the delayed effects of the 2008-09 recession. There is little evidence, however, that recessions in themselves have an impact on inequalities in mortality.^40^ ^41^ ^42^ ^43^ ^44^ A more likely explanation is that this is related to the reductions in public spending that occurred since 2010 as part of the government’s austerity programme. Several recent papers investigating increases in mortality since 2012^45^ ^46^ ^47^ have come to similar conclusions. Other studies have linked recent adverse trends in the health of more disadvantaged groups to cuts in welfare benefits.^48^ ^49^ ^50^ The austerity programme reversed many of the policies that were introduced as part of the strategy^51^ ^52^ ^53^ and we cannot distinguish between the ending of the strategy and the wider programme of austerity that started at the same time (see supplementary appendix 9).

### Implications for policy

Our results have important implications for policy. Whereas previous assessments have concluded that the English health inequalities strategy had little or no effect on geographical health inequalities,^31^ we have found that it was associated with a reduction in geographical inequalities, reversing a long term adverse trend. The findings indicate that a comprehensive strategy characterised by an increase in social investment targeted at the most deprived parts of the country, in conjunction with high level commitment from across government departments backed up by national targets, could be effective at reducing geographical heath inequalities.^10^ This approach has, however, been disbanded by the governments in power since 2010 and it is of particular concern that we are now seeing a reversal of the gains made during the strategy period. In her first speech in July 2016, the UK’s new Prime Minister, Theresa May, stressed her desire to tackle health inequalities. To do this she will need to build on and learn from the successes of the previous health inequalities strategy, rather than following a policy programme that may further widen health inequalities.

**What is already known on this topic**

Between 1997 and 2010 the UK government implemented a comprehensive strategy to reduce health inequalities in England
The Department of Health’s own assessment in 2010 concluded that the targets of the strategy had not been met, and several commentators concluded that it had not been successful
The effects of the strategy, however, may not have been fully realised by this time, and more recent studies have reported that inequalities in mortality narrowed during the strategy period

**What this study adds**

Trends in geographical health inequalities before, during, and after the strategy show that the strategy may have reduced these inequalities, reversing a previously increasing trend
The findings suggest that a cross government strategy that targets increased social investment at more deprived areas and population groups can reduce health inequalities
Current government policies are potentially reversing these gains, and future approaches should learn from the experience of the 1997-2010 strategy

## Web extra

Extra material supplied by authors

**Supplementary information:** appendices 1-14

## Notes

We thank Tom Hennell for his support and advice on initial drafts of this paper and Rebecca Holley from the Office for National Statistics for her help with accessing the mortality data.

Ethical approval: Not required.

Data sharing: Statistical code and data are available from the corresponding author.

Transparency: The lead author (BB) affirms that the manuscript is an honest, accurate, and transparent account of the study being reported; that no important aspects of the study have been omitted; and that any discrepancies from the study as planned have been explained.

## References

[1] House of Commons Health Committee. Health inequalities: Third Report of Session 2008-09. Volume 1. Hc286-1. London: The Stationery Office, 2009.

[2] Mackenbach JP. Can we reduce health inequalities? An analysis of the English strategy (1997-2010). J Epidemiol Community Health 2011;65:568-75. 10.1136/jech.2010.128280 pmid:21459930. 21459930

[3] National Audit Office. Tackling inequalities in life expectancy in areas with the worst health and deprivation. London: NAO.

[4] Acheson SD, Barker D, Chambers J, Graham H, Marmot M, Whitehead M. Independent Inquiry Into Inequalities in Health: Report.Stationery Office, 1998.

[5] Department of Health. Reducing health inequalities: an action report. 1999; published online Jan 1. http://webarchive.nationalarchives.gov.uk/+/www.dh.gov.uk/en/Publicationsandstatistics/Publications/PublicationsPolicyAndGuidance/DH_4006054 (accessed Jan 7, 2015).

[6] Department of Health. Tackling health inequalities: A Programme for Action. London: Health Inequalities Unit, 2003 http://webarchive.nationalarchives.gov.uk/20120804220702/http://www.dh.gov.uk/en/Publicationsandstatistics/Publications/PublicationsPolicyAndGuidance/DH_4008268.

[7] Department of Health. Tackling health inequalities: 2007 Status Report on the Programme for Action. London: Health Inequalities Unit, 2008 http://webarchive.nationalarchives.gov.uk/20130123193251/http://www.dh.gov.uk/en/Publicationsandstatistics/Publications/PublicationsPolicyAndGuidance/DH_083471 (accessed March 12, 2012).

[8] Barr B, Bambra C, Whitehead M. The impact of NHS resource allocation policy on health inequalities in England 2001-11: longitudinal ecological study. BMJ 2014;348:g3231-3231. 10.1136/bmj.g3231 pmid:24865459. 24865459 PMC4035504

[9] Treasury HM. Departmental Report of the Chancellor of the Exchequer’s Departments - GOV.UK. London, 2001 https://www.gov.uk/government/publications/departmental-report-of-the-chancellor-of-the-exchequers-departments (accessed April 20, 2017).

[10] Office of the Deputy Prime Minister. Local Area Agreements Guidance.Stationery Office, 2006.

[11] Barr B, Taylor-Robinson D, Whitehead M. Impact on health inequalities of rising prosperity in England 1998-2007, and implications for performance incentives: longitudinal ecological study. BMJ 2012;345:e7831 10.1136/bmj.e7831 pmid:23212879. 23212879 PMC3514473

[12] Department of Health. Tackling health inequalities: 10 years on a review of developments in tackling health inequalities in England over the last 10 years. London: Department of Health, 2009 http://dera.ioe.ac.uk/11036/ (accessed Oct 15, 2014).

[13] Joyce R, Sibieta L. An assessment of Labour’s record on income inequality and poverty. Oxf Rev Econ Policy 2013;29:178-202 10.1093/oxrep/grt008.

[14] Robinson S, Bugler C. Smoking and drinking among adults, 2009.ONS, 2010.

[15] Scholes S, Bajekal M, Love H, et al. Persistent socioeconomic inequalities in cardiovascular risk factors in England over 1994-2008: a time-trend analysis of repeated cross-sectional data. BMC Public Health 2012;12:129 10.1186/1471-2458-12-129 pmid:22333887. 22333887 PMC3342910

[16] Mackenbach JP. The English strategy to reduce health inequalities. Lancet 2011;377:1986-8. 10.1016/S0140-6736(10)62055-7 pmid:21074842. 21074842

[17] Department of Health. Mortality Target Monitoring.Department of Health, 2009.

[18] ONS. Explaining the Difference between the 2011 Census Estimates and the Rolled-Forward Population Estimates.Office for National Statistics, 2012.

[19] Barr B, Bambra C, Whitehead M. The impact of NHS resource allocation policy on health inequalities in England 2001-11: longitudinal ecological study. BMJ 2014;348:g3231 10.1136/bmj.g3231 pmid:24865459. 24865459 PMC4035504

[20] Buck D, Dixon A. Improving the allocation of health resources in England.Kings Fund, 2013.

[21] Asaria M, Ali S, Doran T, et al. How a universal health system reduces inequalities: lessons from England. J Epidemiol Community Health 2016;70:637-43.pmid:26787198. 26787198 10.1136/jech-2015-206742 PMC4941190

[22] Trend in life expectancy at birth and at age 65 by socio-economic position based on the National Statistics Socio-economic Classification, England and Wales - Office for National Statistics. https://www.ons.gov.uk/peoplepopulationandcommunity/birthsdeathsandmarriages/lifeexpectancies/bulletins/trendinlifeexpectancyatbirthandatage65bysocioeconomicpositionbasedonthenationalstatisticssocioeconomicclassificationenglandandwales/2015-10-21 (accessed March 23, 2017).

[23] Hu Y, van Lenthe FJ, Judge K, et al. Did the English strategy reduce inequalities in health? A difference-in-difference analysis comparing England with three other European countries. BMC Public Health 2016;16:865 10.1186/s12889-016-3505-z. pmid:27558269. 27558269 PMC4995654

[24] Office of National Statistics. SN 3625 Local Mortality Datapack: Population and Deaths by Cause, 1979-1992. https://discover.ukdataservice.ac.uk/catalogue/?sn=3625&type=data%20catalogue (accessed Dec 22, 2014).

[25] Drever F, Fisher K, Brown J, Clark J, Hill S. Social inequalities. HM Stationery Office, 2000 https://www.ons.gov.uk/ons/rel/social-inequalities/focus-on-social-inequalities/2000-edition/social-inequalities.pdf (accessed March 14, 2017).

[26] Great Britain. Neighbourhood Renewal Unit, Great Britain. Office of the Deputy Prime Minister. The English indices of deprivation 2004.Office of the Deputy Prime Minister, 2004.

[27] Life Expectancy at Birth and at Age 65 by Local Areas in the United Kingdom - Office for National Statistics. http://www.ons.gov.uk/peoplepopulationandcommunity/birthsdeathsandmarriages/lifeexpectancies/bulletins/lifeexpectancyatbirthandatage65bylocalareasintheunitedkingdom/2014-04-16 (accessed April 11, 2016).

[28] ONS. NOMIS–official labour market statistics. 2014. http://www.nomisweb.co.uk/ (accessed Sept 10, 2014).

[29] Crawley MJ. The R Book.Wiley-Blackwell, 2007 10.1002/9780470515075.

[30] Kunst AE, Bos V, Mackenbach J. Monitoring socio-economic inequalities in health in the European Union: guidelines and illustrations.Erasmus University, 2001.

[31] The Marmot Review. Fair Society.Healthy Lives, 2010.

[32] Marmot M. World Health Organization, UCL Institute of Health Equity, editors. Review of social determinants and the health divide in the WHO European Region: final report. Copenhagen: World Health Organization, Regional Office for Europe, 2014.

[33] Driscoll J, Kraay A. Consistent Covariance Matrix Estimation With Spatially Dependent Panel Data. Rev Econ Stat 1998;80:549-60 10.1162/003465398557825.

[34] Hoechle D. others. Robust standard errors for panel regressions with cross-sectional dependence. Stata J 2007;7:281.

[35] Buck D, Maguire D. Inequalities in life expectancy. *Chang Time Implic Policy Kings Fund* 2015. http://www.commed.vcu.edu/IntroPH/Community_Assessment/2015/ukequityf15.pdf (accessed July 22, 2016).

[36] Maheswaran H, Kupek E, Petrou S. Self-reported health and socio-economic inequalities in England, 1996-2009: Repeated national cross-sectional study. Soc Sci Med 2015;136-137:135-46.pmid:26004207. 26004207 10.1016/j.socscimed.2015.05.026 PMC4510149

[37] Katikireddi SV, Niedzwiedz CL, Popham F. Trends in population mental health before and after the 2008 recession: a repeat cross-sectional analysis of the 1991-2010 Health Surveys of England. BMJ Open 2012;2:e001790 10.1136/bmjopen-2012-001790. pmid:23075569. PMC3488736 23075569

[38] Dixon A, Khachatryan A, Gilmour S. Does general practice reduce health inequalities? Analysis of quality and outcomes framework data. Eur J Public Health 2012;22:9-13. 10.1093/eurpub/ckq177. pmid:21148179. 21148179

[39] Hippisley-Cox J. Health Inequalities in Primary Care: Effect of Spearhead Primary Care Trusts 2002-2009. National Audit Office http://www.nao.org.uk/report/tackling-inequalities-in-life-expectancy-in-areas-with-the-worst-health-and-deprivation/ (accessed March 10, 2014).

[40] Parmar D, Stavropoulou C, Ioannidis JPA. Health outcomes during the 2008 financial crisis in Europe: systematic literature review. BMJ 2016;354:i4588 10.1136/bmj.i4588 pmid:27601477. 27601477 PMC5013230

[41] Valkonen T, Martikainen P, Jalovaara M, Koskinen S, Martelin T, Mäkelä P. Changes in socioeconomic inequalities in mortality during an economic boom and recession among middle-aged men and women in Finland. Eur J Public Health 2000;10:274-80 10.1093/eurpub/10.4.274.

[42] Tapia-Granados J. Recessions and mortality in Spain, 1980-1997. Eur J Popul 2005;21:393-422 10.1007/s10680-005-4767-9.

[43] Bartoll X, Toffolutti V, Malmusi D, Palència L, Borrell C, Suhrcke M. Health and health behaviours before and during the Great Recession, overall and by socioeconomic status, using data from four repeated cross-sectional health surveys in Spain (2001-2012). BMC Public Health 2015;15:865 10.1186/s12889-015-2204-5 pmid:26346197. 26346197 PMC4561448

[44] Suhrcke M, Stuckler D. Will the recession be bad for our health? It depends. Soc Sci Med 2012;74:647-53. 10.1016/j.socscimed.2011.12.011 pmid:22226605. 22226605

[45] Hiam L, Dorling D, Harrison D, McKee M. Why has mortality in England and Wales been increasing? An iterative demographic analysis. J R Soc Med 2017;110:153-62.pmid:28208027. 28208027 10.1177/0141076817693599 PMC5407517

[46] Hiam L, Dorling D, Harrison D, McKee M. What caused the spike in mortality in England and Wales in January 2015?J R Soc Med 2017;110:131-7. 10.1177/0141076817693600 pmid:28208024. 28208024 PMC5407518

[47] Green M, Dorling D, Minton J. The Geography of a rapid rise in elderly mortality in England and Wales, 2014-15. Health Place 2017;44:77-85. 10.1016/j.healthplace.2017.02.002 pmid:28199896. 28199896

[48] Barr B, Kinderman P, Whitehead M. Trends in mental health inequalities in England during a period of recession, austerity and welfare reform 2004 to 2013. Soc Sci Med 2015;147:324-31. 10.1016/j.socscimed.2015.11.009 pmid:26623942. 26623942

[49] Barr B, Taylor-Robinson D, Stuckler D, Loopstra R, Reeves A, Whitehead M. ‘First, do no harm’: are disability assessments associated with adverse trends in mental health? A longitudinal ecological study. J Epidemiol Community Health 2016;70:339-45.pmid:26573235. 26573235 10.1136/jech-2015-206209 PMC4819657

[50] Loopstra R, McKee M, Katikireddi SV, Taylor-Robinson D, Barr B, Stuckler D. Austerity and old-age mortality in England: a longitudinal cross-local area analysis, 2007-2013. J R Soc Med 2016;109:109-16. 10.1177/0141076816632215 pmid:26980412. 26980412 PMC4794969

[51] Barr B, Taylor-Robinson D. Poor areas lose out most in new NHS budget allocation. BMJ 2014;348:g160-160. 10.1136/bmj.g160 pmid:24429924. 24429924

[52] Taylor-Robinson D, Gosling R. English north-south divide. Local authority budget cuts and health inequalities. BMJ 2011;342:d1487-1487. 10.1136/bmj.d1487 pmid:21385816. 21385816

[53] Taylor-Robinson D, Whitehead M, Barr B. Great leap backwards. BMJ 2014;349:g7350-7350. 10.1136/bmj.g7350 pmid:25468834. 25468834

[54] House of Commons Health Committee. Public health post–2013. London: House of Commons, 2017.

