# Supplementary material for: Investigating the impact of the English health inequalities strategy: time trend analysis
Source: BMJ. 2017 Jul 26;358:j3310. doi: 10.1136/bmj.j3310 (PMC5527348; doi:10.1136/bmj.j3310)

Web Appendices - Investigating the impact of the English Health Inequalities Strategy: a time trend analysis.

Appendix 1. Elements of the English Health Inequalities Strategy and progress against the 82 Departmental Commitments. 2

Appendix 2. Model formula 9

Appendix 3. Group specific trend estimates derived from the regression models. 10

Appendix 4. Alternative models 11

Appendix 5. Simulation study investigating likely errors that would result from using a comparison between Spearhead and non-Spearhead areas to identify a change in the trend in spatial socioeconomic inequalities. 23

Appendix 6. Trend in life expectancy in Spearhead and non-Spearhead areas 26

Appendix 7. Identifying 'natural' breakpoints in the trend in inequalities. 28

Appendix 8. Comparing the change in inequalities across neighbourhoods *within* the most deprived local authorities and the rest of England. 30

Appendix 9. Trend in inequalities, poverty measures and government expenditure. 31

Appendix 10. Analysis showing the effect of population revisions on the gap in life expectancy between the most deprived LAs and the rest of England between 2006-2010. 36

Appendix 11. Trend in life expectancy and descriptive data for the deprived group of local authorities and the rest of England. 37

Appendix 12. Age specific trends in inequalities. 39

Appendix 13. Relative change in deaths under 65. 40

Appendix 14. Investigating the non-linear relationship between deprivation and increase in life expectancy before, during and after the strategy 41

Appendix 1. Elements of the English Health Inequalities Strategy and progress against the 82 Departmental Commitments.

- 1998 - Acheson Independent Inquiry into Inequalities in Health.
- 1998 – First wave of Health Action Zones established in disadvantaged areas to bring together public, private and voluntary organisations to reshape local health and social services and to improve the health of their local populations.
- 1998 – First Sure Start children's centres established in deprived areas to improve provision of childcare, early education, health and family support.
- 1998 - New Deal for Communities – an area based regeneration initiative targeting 39 disadvantaged areas in England.
- 1998 - National Minimum Wage Act introduced the first minimum wage in the UK
- 1999 - Government targets announced to 'eradicate' child poverty by 2020–21, along with interim child poverty targets for 2004–05 and 2010–11.
- 1999 - Department of Health issued "Reducing Health Inequalities: an Action Report" setting out national actions to be taken in response to the Acheson Report including tackling low income, tax and benefit changes and improving early education through the establishment of Sure Start centres.
- 1999 - A new objective introduced for the allocation of resources in the NHS in England 'to contribute to the reduction in avoidable health inequalities'.
- 2002 - Tackling Health Inequalities: Cross-Cutting Review - set infant mortality and life expectancy targets.
- 2002 - New health inequalities component introduced to the NHS resource allocation formula.
- 2003 - Cross-government health inequalities strategy, A Programme for Action, included 12 cross-government headline indicators and 82 cross-government commitments targeted towards low-income groups or deprived areas, including poverty reduction, improved education, expansion of the Sure Start scheme, expansion of smoking cessation services, improvement of primary care facilities and improved access to treatment for cancer and cardiovascular disease.
- 2004 - *Choosing Health: Making Healthy Choices Easier* – introduced a number of initiatives to reduce smoking, obesity, increase exercise, alcohol misuse and improve sexual and mental health.
- 2004 - *Child Poverty Review* – setting out policies to improve employment opportunities, increase support for those who cannot work, improve housing and reduce homelessness, improve education and services for children and their families in deprived areas.
- 2005 - Life expectancy target refined to define a fixed group of Spearhead Local Authorities with the worst health and deprivation indicators.
- 2006 - *NHS Operating Framework* established health inequalities as one of six top NHS priorities and Spearhead areas were obliged to report on progress they were making in reducing health inequalities through local delivery plans.
- 2007- Health Inequalities National Support Team established to share good practice, and enable local areas to understand the observed gaps in life expectancy and evidenced based approaches which could have a rapid impact (6).
- 2008- Health inequalities introduced as a key performance indicator for the NHS (known as Vital Sign indicators) with performance explicitly managed by strategic health authorities.

Summary of progress against 82 departmental commitments (Source: Department of Health. "Tackling Health Inequalities: 10 Years on a Review of Developments in Tackling Health Inequalities in England over the Last 10 Years." London: Department of Health, 2009).

The *Programme for Action* identified 82 cross-departmental commitments to support the national strategy by 12 government departments, chiefly the Department of Health, the Department for Children, Schools and Families, Communities and Local Government and the Department for Work and Pensions. For the most part, these commitments covered the period 2003–06. A summary of the progress, included below shows that 75 out of 82 commitments (91%) had been wholly or substantially achieved, by December 2006.

		Owner	Status
	**SUPPORTING FAMILIES, MOTHERS AND CHILDREN**		
	**Maternal and child health, and child development**		
	Support poorer families and children by:		
1	– expanding Sure Start services for children under 6 and their families, Sure Start local programmes to reach 400,000 children living in disadvantaged areas, including a third of children under 4 living in poverty	DCSF	Green
2	– developing a network of Children's Centres in 20% of the most disadvantaged wards, reaching up to 650,000 children and their families	DCSF	Green
3	– creating a further 250,000 new childcare places by 2006, 180,000 in the 20% of most disadvantaged wards	DCSF	Green
4	– establishing 45,000 new daycare places through the Neighbourhood Nursery Initiative	DCSF	Green
5	– providing free nursery education for all 3-year-olds	DCSF	Green
6	– ensuring that 800,000 children, pregnant women and mothers from low-income families have a healthy diet through the reformed Welfare Food Scheme and provide better support for breastfeeding mothers.	DH	Green
	**Improving life chances for children and young people**		
7	Support children and young people at risk aged 5–13 through the Children's Fund, with spend of £150 million for each of three years to 2006.	DCSF	Green
8	Address mental health needs of children by establishing a comprehensive child and adolescent mental health service (CAMHS) in all areas by 2006, with an extra £250 million to 2006.	DH	Amber/ Green
9	Improve the quality of life of marginalised young people using sport to raise their aspirations and connect them back to education, training and employment through Positive Futures projects.	HO	Green
10	Develop and improve sports facilities for around 2,300 schools and raise standards of physical education in disadvantaged areas.	DCMS DCSF	Green
11	Expand the specialist sports college and School Sport Co-ordinator Programmes to create a network of 400 school sports co-ordinator partnerships. Spend of £339 million to 2006.	DCSF, DCMS	Green
	Meet the needs of disadvantaged individuals, groups and areas at school through mainstream education services and targeted action by 2004, specifically by:		
12	– establishing a GCSE floor target to ensure that 25% of pupils in every school gain five A*–C GCSEs	DCSF	Amber

		Owner	Status
13	– improving education of children in care to substantially narrow the gap between educational attainment and participation of their peers by 2006	DCSF	Amber
14	– improving behaviour and school attendance in the worst areas through the £470 million National Behaviour and Attendance programme	DCSF	**Green**
15	– improving learning outcomes for pupils in disadvantaged areas through the Creative Partnership programme. Spend £27 million in 2003/04 rising to £45 million in 2005/06	DCSF, DH	**Green**
16	– improving the social and health context of school life by targeting the Healthy Schools programme on the most deprived communities	DCSF	**Green**
17	– reducing the number of 16–18-year-olds not engaged in education, employment or training by 10% in established Connexion partnerships.	DCSF	**Green**
	**Reducing teenage pregnancy and supporting teenage mothers**		
18	Raise the quality of education in schools by the introduction of a certification programme on sex and relationship education for teachers and equivalent programmes for school and community nurses.	DCSF	**Green**
19	Share learning and best practice from the Sure Start Plus teenage pregnancy pilot programmes with Connexions personal advisers and others.	DCSF	**Green**
20	Improve access for young parents to antenatal and postnatal care.	DCSF, DH	**Amber/ Green**
21	Improve access to learning and employment opportunities through the Connexions programme.	DCSF	**Amber/ Green**
	**ENGAGING COMMUNITIES AND INDIVIDUALS**		
22	Continue to support the reshaping and redirecting of mainstream services to tackle the problems faced in disadvantaged neighbourhoods through the National Strategy for Neighbourhood Renewal supported by the Neighbourhood Renewal Fund.	CLG	**Green**
23	Encourage greater communities involvement in actions to improve the local environments and make them healthier places to be.	CLG	**Green**
24	Support existing and new health initiatives through further investment to PCTs in Health Action Zone areas to 2006.	DH	**Green**
25	Use schools to improve services for local people through the creation of up to 240 full-service Extended Schools by 2006, targeted initially at areas of deprivation and offering a set of services including health and social care, childcare adult education and sports activities.	DCSF	**Green**
26	Deliver services for 'hard to reach' groups through the 257 healthy living centres clustered round areas of deprivation from 2003.	DH	**Green**
27	Support vulnerable groups through the Supporting People programme, including teenage parents, victims of domestic violence and ex-offenders, as well as independent living within communities for older, disabled and vulnerable people.	CLG	**Green**
	**Enterprise**		
28	Promote the provision of business support and finance for entrepreneurs from disadvantaged groups through the Phoenix Fund and the work of the Regional Development Agencies.	BERR	**Green**
29	Encourage community-based enterprises to provide services to the public sector through the development of a 'good corporate citizen' approach in the NHS and local authorities.	DH, CLG, BERR	**Green**

		Owner	Status
	**Crime/drug misuse**		
30	Increase participation of problem users in treatment programmes, maintain the proportion successfully completing treatment programmes, further expand the drug treatment workforce, and improve access to treatment programmes, driving down the waiting lists across all treatments.	DH/HO	Green
	**Older people**		
31	Involve older people in both high-level policy direction at a national level of policy, and service development at a local level outlined in the National Service Framework (NSF) for Older People.	DH	Green
32	Improving access to, the effectiveness of, and the integration of, falls prevention services through the direct involvement of older people and their representative organisations in local health communities and falls collaborative actions.	DH	Green
	**Homeless people**		
	Tackle and prevent homelessness through homelessness strategies and meeting the Government's targets to:		
33	– ensure no homeless family with children is in bed and breakfast accommodation by March 2004, unless for urgent cases and even then no longer than for six weeks	CLG	Green
34	– sustain or reduce the numbers of people sleeping rough at 600 people or fewer.	CLG	Green
	**People with mental Illness**		
35	Reduce the duration of untreated psychosis to three months by 2004 by establishing intervention teams and provide support for the first three years for all young people who develop an episode of psychosis.	DH	Green
36	Provide access crisis resolution services from 2005, either from the teams or trained NHS Direct staff.	DH	Green
	**Prisoners' health**		
37	Address prisoners' mental health needs by providing all prisoners with severe mental health problems with a care plan by 2004.	DH	Green
	**Asylum seekers and refugees**		
38	Assess health needs through a network of induction centres, all of which will include the provision of a health assessment.	DH	Green
39	Meet the language needs of this group through developing an online resource of health information in key languages and a national scoping study on models of providing interpreting services for NHS Direct.	DH	Green
	**PREVENTING ILLNESS AND PROVIDING EFFECTIVE TREATMENT AND CARE**		
	**Reducing risk through effective prevention**		
	Reduce smoking, particularly among manual groups by:		
40	– expanding PCT smoking cessation services	DH	Green
41	– expanding tailored tobacco education campaigns, for example in prisons, hospitals and factories	DH	Green
42	– ending tobacco advertising, promotion and sponsorship	DH	Green
43	– running extended mass media education campaigns	DH	Green
44	– enforcing a ban on under-age sales of tobacco	DH	Green

		Owner	Status
45	– putting new health warnings and advice on tobacco products to achieve 800,000 quitters at the four-week stage by 2006, and reducing smoking in pregnancy by 1 percentage point a year 2003–06.	DH	**Green**
	Improve diet and nutrition among disadvantaged groups and children by implementing the Food and Health Action Plan across Government and other sectors and:		
46	– further develop the 5 A DAY programme targeting both the 66 PCTs in the most deprived areas of the country funded by the New Opportunities Fund until 2005, and in disadvantaged areas in all PCTs	DH	**Green**
47	– expanding the National School Fruit Scheme to reach all children aged 4–6 by 2004.	DH	**Green**
48	Increase participation in physical activity through the introduction of Local Exercise Action Pilots.	DH, DCMS	**Green**
49	Reduce accidental injury, especially among children and young people in disadvantaged areas, through the environmental improvements, public education campaigns and projects to reduce child road casualties.	DfT, HO, DCSF	**Green**
50	Reduce deaths and injuries from house fires through national awareness campaigns and targeted fire service risk-management strategies.	CLG	**Green**
51	Develop co-ordinated local action programmes that improve the health and wellbeing of older people through the NSF for Older People.	DH	**Amber**
	**Early detection, intervention and treatment**		
52	Increase resources available to the NHS to take account of unmet need through the new NHS resource allocation formula and devolved PCT budgets.	DH	**Green**
53	Improve primary care facilities, especially in inner cities and urban areas, by £1 billion programme of refurbishing or replacing 3,000 family doctor's premises and establishing 500 one-stop centres.	DH	**Green**
54	Raise the quality of service in disadvantaged areas by establishing 20 teaching PCTs by 2004.	DH	**Green**
55	Improve access to rural services by establishing 100 one-stop primary care centres or mobile service units by 2004.	DH	**Green**
	Further improve mainstream primary care services by:		
56	– providing guaranteed access to a primary care professional within one working day, and to a GP within two working days by December 2004. NHS Walk-In Centres are one of the services available to PCTs to improve access to primary care	DH	**Amber/ Green**
57	– creating coronary heart disease (CHD) practice-based patient registers to ensure systematic treatment regimes for those at most risk by March 2006	DH	**Green**
58	– extending breast cancer screening to women aged 65–70 by 2004 and agreeing local protocols to address inequalities in service provision	DH	**Amber/ Green**
59	– meeting the target of 70% uptake in flu immunisation in people aged 65 years and over, especially in areas of lowest life expectancy	DH	**Green**
60	– quality assuring screening programmes to ensure uptake is equitable and reaching those most in need.	DH	**Amber/ Green**
	Implement the NSF for Older People by:		
61	– supporting action to identify and eliminate age discrimination in access to health and social care	DH	**Amber/ Green**
62	– developing a local Single Assessment Process with shared information and assessment mechanisms across health and social care covering stroke care, falls services and mental health.	DH	**Green**

		Owner	Status
	**Improving access to effective treatment**		
63	Respond to local needs and raise standards of service through NHS Foundation Trusts – hospitals in some of the most deprived areas have expressed interest in being among the first trusts, and all hospitals will be given help to become a trust over the course of the next four to five years.	DH	**Green**
64	Improve access to health facilities by PCTs working in partnership with local authority transport planners to conduct accessibility planning, reform of patient transport services and the hospital travel costs scheme.	DH	**Green**
65	Improve access to cancer services by treating all cancer patients within a month of diagnosis and within two months of urgent referral by 2005.	DH	**Amber**
66	Improve access to CHD services by setting a two-week wait standard for rapid access chest pain clinics and a three-month maximum wait for angiography and revascularisation by 2005.	DH	**Green**
67	Promote rehabilitation and supported discharge from hospital with 150,000 additional people receiving intermediate care services by March 2004.	DH	**Green**
	**ADDRESSING THE UNDERLYING DETERMINANTS OF HEALTH**		
	**Child poverty**		
68	Reduce the number of children in low-income households by a quarter by 2004/05 from 1998/99 as a contribution to the broader target of halving child poverty by 2010 and eradicating it by 2020.	DWP, HMT	**Amber**
	**Housing and environment**		
69	Improve the quality of social housing and raise 370,000 homes above the decent homes standard by 2006.	CLG	**Green**
70	Address the needs of poor households in the private sector and raise 80,000 households to the decent homes standard by 2005/06.	CLG	**Green**
71	Eradicate fuel poverty in England among vulnerable households by 2010 and by 2016 for all other households as far as reasonably practicable.	Defra, BERR	**Amber/ Red**
72	Reduce fuel poverty by improving the energy efficiency of homes for 800,000 vulnerable households through the Warm Front programme by 2004.	Defra	**Green**
73	Work for cleaner, safer and greener local environments and thriving sustainable communities through *Living Places: Cleaner, Safer, Greener and Sustainable Communities: building for the future.*	CLG	**Green**
	**Training and skills**		
74	Improve the basic skills of 750,000 adults through the continued expansion of the Skills for Life programme by 2004.	DCSF	**Green**
75	Develop and deliver literacy, numeracy and English language training for 20,000 health and social care staff through the NHS University by 2006.	DH	Amber
	**Jobs and income**		
76	Enable people with health problems and disabilities to move into work through the Pathways to Work programme.	DWP	**Green**
77	Provide extra support for people in work, families and older people through the working and child tax credits, and pension credit.	DWP, HMT	**Green**
78	Help people who are unemployed (but available to work) to return to the labour market through Jobcentre Plus. The implementation of new-style Jobcentre Plus offices throughout its local office network to be complete by 2006.	DWP	**Green**

		Owner	Status
	**Transport**		
79	Oversee the implementation of the Social Exclusion Unit action plan to improve access to jobs and key services, to March 2005.	DfT	**Amber/ Green**
80	In areas that produce Local Transport Plans, transport planners will lead work to improve access to jobs and key services. This process, accessibility planning, will be incorporated into authorities' second Local Transport Plans by 2005.	DH/DfT	**Amber/ Green**
81	Encourage more children to walk and cycle, through a package of measures to promote sustainable travel to school.	DfT	**Green**
82	Forthcoming changes to the bus registration system will make it easier to register flexibly routed, demand-responsive services. This will allow the provision of dedicated door-to-door bus services tailored to meet passenger needs.	DfT	**Green**

KEY

Green	**GREEN** – Commitment achieved in full and on time, or currently on target to deliver on time
Amber/Green	**AMBER/GREEN** – Commitment substantially achieved, but not full coverage or slightly late
Amber	**AMBER** – Commitment partly achieved or substantially delayed
Amber/Red	**AMBER/RED** – Commitment mostly not achieved, but action has some impact
Red	**RED** = Commitment not achieved
Black	**BLACK** = No response

Appendix 2. Model formula

We estimated whether the strategy period was associated with a greater decline in absolute inequalities between the most deprived local authorities and the rest of England compared to the before and after periods using segmented linear regression, controlling for the trend in unemployment. Specifically, we estimated the following model:

Equation 1: $LE_{i,t} = \beta_1 t_1 + \beta_2 Deprived + \beta_3 Deprived * t_1 + \beta_4 t_2 + \beta_5 Deprived * t_2 + \beta_6 t_3 + \beta_7 Deprived * t_3 + \beta_8\, Unemp_{i,t} + u_i + \varepsilon_{it}$

Where $LE_{i,t}$ is the life expectancy in LA i in year t,

t_1 is annual trend term for the before period

t_2 is annual trend term for the strategy period

t_3 is annual trend term for the after period

Deprived is a dummy variable indicating whether an LA is within the most deprived quintile. Unemp is the annual unemployment rate in each LA as measured as the proportion of 16-64 year olds claiming of unemployment benefits. u_i is a fixed effect for each local authority. The breakpoints defining the beginning and end of the strategy period were defined based on the process outlined in Appendix 7.

Appendix 3. Group specific trend estimates derived from the regression models.
Table A1. Trend in life expectancy in the most deprived LAs and the rest of the country - before, during and after the health inequalities strategy. Trend is shown as the annual change in life expectancy measured in months.

**Men**	Annual change (in months) in life expectancy	95%CI		P-value for trend	P-value for change in trend from previous time period
**Deprived areas**					
Before (1983-2003)	2.35	2.17	2.53	<0.001	
During (2004-2012)	4.90	4.54	5.26	<0.001	<0.001
After (2013-2015)	-0.78	-1.61	0.05	0.06	<0.001
**The rest of the country**					
Before (1983-2003)	2.92	2.86	2.99	<0.001	
During (2004-2012)	4.00	3.88	4.11	<0.001	<0.001
After (2013-2015)	-0.10	-0.42	0.22	0.5	<0.001
**Women**	Annual change (in months) in life expectancy	95%CI		P-value for trend	P-value for change in trend from previous time period
**Deprived areas**					
Before (1983-2003)	1.69	1.51	1.87	<0.001	
During (2004-2012)	3.58	3.23	3.93	<0.001	<0.001
After (2013-2015)	-1.01	-1.53	-0.49	<0.001	<0.001
**The rest of the country**					
Before (1983-2003)	1.99	1.92	2.06	<0.001	
During (2004-2012)	3.08	2.96	3.2	<0.001	<0.001
After (2013-2015)	-0.7	-0.98	-0.42	<0.001	<0.001

Appendix 4. Alternative models

Table A2. Trend in relative inequalities in life expectancy, between the most deprived LAs and the rest of the country before, during and after the health inequalities

strategy. Trend is shown as the annual increase (+) or decrease (-) in the percentage difference in life expectancy between deprived LAs and the rest of the country.

**Men**	Annual change in the relative percentage gap in life expectancy between the most deprived 20% of local authorities and the rest of England **[95%CI]**		
Before (1983-2003)	0.056	[0.037,0.075]	<0.001
During (2004-2012)	-0.115	[-0.154,-0.075]	<0.001
After (2013-2015)	0.081	[-0.013,0.175]	0.092
N=10692 LA years, R^2 =0.74			
**Women**			
Before (1983-2003)	0.029	[0.010,0.047]	0.003
During (2004-2012)	-0.059	[-0.095,-0.023]	0.001
After (2013-2015)	0.037	[-0.021,0.095]	0.214
N=10692 LA years, R^2 =0.65			

Note: Estimates based on fixed effects regression model using a local authority panel dataset of life expectancy from 1983 to 2015, also adjusting for local unemployment rates.

Using a continuous measure of deprivation.

To estimate the extent there was a narrowing of inequalities across all levels of deprivation we estimated our models using a continuous measure rather than a binary split between deprived areas and the rest of the country. The IMD 2004 income score was converted to a weighted rank across all local authorities (LA), from the least deprived (0) to the most deprived (1), we then fitted the model including this measure interacted with our linear spline time trend terms. The coefficients of this model can then be interpreted as the absolute annual change in the Slope Index of Inequality[1](i.e the change in the gap between the most deprived and least deprived LAs – assuming a linear relationship between change in inequalities and deprivation).

Table A3. Models using a linear continuous term for deprivation - Trend in absolute inequalities in life expectancy, before, during and after the health inequalities strategy. Trend is shown as the annual increase (+) or decrease (-)change in the Slope Index of Inequality measured in months.

Men	Annual change (in months) in the Slope Index of Inequality life expectancy **[95%CI]**		P-value for trend	P-value for change in trend from previous time period
Before (1983-2003)	1.06	[0.86,1.26]	<0.001	
During (2004-2012)	-1.15	[-1.58,-0.73]	<0.001	<0.001
After (2013-2015)	0.89	[-0.28,2.07]	0.14	<0.001
N=10692 LA years, R^2 =0.79				
**Women**				
Before (1983-2003)	0.61	[0.40,0.81]	<0.001	
During (2004-2012)	-0.32	[-0.74,0.10]	0.14	<0.001
After (2013-2015)	0.34	[-0.45,1.14]	0.40	0.17
N=10692 LA years, R^2 =0.55				

Note: Estimates using a local authority panel dataset of life expectancy from 1983 to 2015, also adjusting for local unemployment rates.

Removing outliers.

Initially we estimated the trend in life expectancy for each local authority between 2004-2012. We then removed all local authorities that had a trend during this period that was +/- 2 standard deviations greater or lesser than the mean (5 deprived LAs and 17 LAs from the rest of England), and re- estimated our model.

Table A4. Removing outliers. Trend in absolute inequalities in life expectancy, between the most deprived LAs and the rest of the country before, during and after the health inequalities strategy. Trend is shown as the annual increase (+) or decrease (-) in the absolute gap in life expectancy measured in months.

Men	Annual change (in months) in absolute gap in life expectancy between the most deprived 20% of local authorities and the rest of England **[95%CI]**		P-value for trend	P-value for change in trend from previous time period
Before (1983-2003)	0.49	[0.32,0.66]	<0.001	
During (2004-2012)	-0.7	[-1.02,-0.38]	<0.001	<0.001
After (2013-2015)	0.73	[-0.18,1.64]	0.11	0.01
N=9966 LA years, R^2 =0.74				
**Women**				
Before (1983-2003)	0.24	[0.06,0.41]	0.01	
During (2004-2012)	-0.35	[-0.68,-0.03]	0.03	0.01
After (2013-2015)	0.32	[-0.26,0.90]	0.28	0.61
N= 10098 LA years, R^2 =0.28				

Table A5. Models without controls for unemployment- Trend in absolute inequalities in life expectancy, between the most deprived LAs and the rest of the country before, during and after the health inequalities strategy. Trend is shown as the annual increase (+) or decrease (-) in the absolute gap in life expectancy measured in months.

Men	Annual change (in months) in absolute gap in life expectancy between the most deprived 20% of local authorities and the rest of England	**[95%CI]**	
Before (1983-2003)	0.54	[0.38,0.69]	<0.001
During (2004-2012)	-0.9	[-1.28,-0.53]	<0.001
After (2013-2015)	0.62	[-0.26,1.51]	0.16
N=10692 LA years, R^2 =0.74			
**Women**			
Before (1983-2003)	0.3	[0.13,0.48]	<0.001
During (2004-2012)	-0.5	[-0.86,-0.15]	0.01
After (2013-2015)	0.32	[-0.26,0.89]	0.28
N=10692 LA years, R^2 =0.66			

Table A6. Models random rather than fixed effects - Trend in absolute inequalities in life expectancy, between the most deprived LAs and the rest of the country before, during and after the health inequalities strategy. Trend is shown as the annual increase (+) or decrease (-) in the absolute gap in life expectancy measured in months.

**Men**	Annual change (in months) in absolute gap in life expectancy between the most deprived 20% of local authorities and the rest of England **[95%CI]**		
Before (1983-2003)	0.6	[0.50,0.70]	0
During (2004-2012)	-0.96	[-1.19,-0.73]	0
After (2013-2015)	0.43	[-0.58,1.45]	0.40
N=10692 LA years, R^2 =0.80			
**Women**			
Before (1983-2003)	0.34	[0.24,0.43]	0
During (2004-2012)	-0.52	[-0.74,-0.30]	0
After (2013-2015)	0.23	[-0.76,1.22]	0.65
N=10692 LA years, R^2 =0.71			

Note: Estimates using a local authority panel dataset of life expectancy from 1983 to 2015, also adjusting for local unemployment rates.

Table A7. Models with alternative breakpoints for the starting point of the strategy - Trend in absolute inequalities in life expectancy, between the most deprived LAs and the rest of the country - before, during and after the health inequalities strategy. Trend is shown as the annual increase (+) or decrease (-) in the absolute gap in life expectancy measured in months. The final breakpoint in each model is fixed at 2012.

**Men**					
**Initial Breakpoint used in model**	Annual change (in months) in absolute gap in life expectancy between the most deprived 20% of local authorities and the rest of England between initial breakpoint and 2012 **[95%CI]**			P-value for trend	P-value for change in trend from previous time period
1997	-0.351	-0.089	-0.613	0.009	<0.001
1998	-0.41	-0.139	-0.681	0.003	<0.001
1999	-0.482	-0.2	-0.764	0.001	<0.001
2000	-0.563	-0.267	-0.859	<0.001	<0.001
2001	-0.664	-0.349	-0.979	<0.001	<0.001
2002	-0.773	-0.436	-1.11	<0.001	<0.001
2003	-0.906	-0.54	-1.272	<0.001	<0.001
2004	-1.068	-0.663	-1.474	<0.001	<0.001
2005	-1.28	-0.824	-1.735	<0.001	<0.001
2006	-1.575	-1.05	-2.101	<0.001	<0.001

Women

Initial Breakpoint used in model	Annual change (in months) in absolute gap in life expectancy between the most deprived 20% of local authorities and the rest of England between initial breakpoint and 2012 [95%CI]			P-value for trend	P-value for change in trend from previous time period
1997	-0.193	0.038	-0.424	0.102	0.012
1998	-0.218	0.025	-0.461	0.079	0.009
1999	-0.254	0.005	-0.513	0.054	0.005
2000	-0.3	-0.021	-0.579	0.035	0.003
2001	-0.357	-0.053	-0.66	0.022	0.002
2002	-0.422	-0.092	-0.752	0.012	0.001
2003	-0.506	-0.142	-0.871	0.007	0.001
2004	-0.613	-0.204	-1.022	0.003	0.001
2005	-0.734	-0.271	-1.198	0.002	<0.001
2006	-0.915	-0.381	-1.45	0.001	<0.001

Table A8. Random effect and random slope. Trend in absolute inequalities in life expectancy, between the most deprived LAs and the rest of the country - before, during and after the health inequalities strategy. Trend is shown as the annual

increase (+) or decrease (-) in the absolute gap in life expectancy measured in months.

**Men**	Annual change (in months) in absolute gap in life expectancy between the most deprived 20% of local authorities and the rest of England **[95%CI]**		
Before (1983-2003)	0.63	[0.44,0.82]	<0.001
During (2004-2012)	-0.96	[-1.17,-0.76]	<0.001
After (2013-2015)	0.48	[-0.43,1.39]	0.30
N=10692 LA years			
**Women**			
Before (1983-2003)	0.36	[0.18,0.54]	<0.001
During (2004-2012)	-0.52	[-0.73,-0.32]	<0.001
After (2013-2015)	0.27	[-0.63,1.17]	0.55
N=10692 LA years			

Note: Estimates using a local authority panel dataset of life expectancy from 1983 to 2015, also adjusting for local unemployment rates.

Table A9. Fixed effects models with Driscoll and Kraay (1998) standard errors for autocorrelation. Trend in absolute inequalities in life expectancy, between the most

deprived LAs and the rest of the country before, during and after the health inequalities strategy. Trend is shown as the annual increase (+) or decrease (-) in the absolute gap in life expectancy measured in months.

As there was evidence of autocorrelation in the time series, we initially estimated the maximum lags required to take into account the autocorrelation structure using Newey and West's (1994) automatic bandwidth selection procedure.[2] This indicated a maximum lag of 16 was appropriate.

**Men**	Annual change (in months) in absolute gap in life expectancy between the most deprived 20% of local authorities and the rest of England **[95%CI]**		
Before (1983-2003)	0.56	[0.49,0.62]	<0.001
During (2004-2012)	-0.95	[-1.15,-0.75]	<0.001
After (2013-2015)	0.36	[-0.13,0.86]	0.15
N=10692 LA years, R2 86			
**Women**			
Before (1983-2003)	0.31	[0.26,0.36]	<0.001
During (2004-2012)	-0.51	[-0.68,-0.35]	<0.001
After (2013-2015)	0.18	[-0.28,0.64]	0.45
N=10692 LA years			

Note: Estimates using a local authority panel dataset of life expectancy from 1983 to 2015, also adjusting for local unemployment rates.

Table A10. Controlling for trends in migration. Trend in absolute inequalities in life expectancy, between the most deprived LAs and the rest of the country before, during

and after the health inequalities strategy. Trend is shown as the annual increase (+) or decrease (-) in the absolute gap in life expectancy measured in months.

We only investigated change in inequalities at the area level. It is possible that the observed trends in health inequalities are due to a change in the composition of the populations in those areas, rather than a reduction in inequalities in individual mortality risks. To investigate this we estimate whether the declining trend in health inequalities during the strategy period changed when we adjusted for differential trends in migration.

Data were only available on migration at the local authority level from the ONS for the years 2004 to 2014. To investigate whether migration patterns were likely to influence our results we estimated further models limited to this period with time trend terms for the strategy period (2004-2012) and the period following the strategy (2013-2014). We calculated migration inflow and outflow rates for international and internal migration for each local authority in each year using migration flows data from the ONS.[3] We then estimated models without (A) and with (B) controls for migration to investigate whether controlling for migration changed the estimate of the trend in health inequalities during the strategy period.

These show that adjusting for migration patterns did not affect the trend in inequalities during the strategy period.

A. **Estimates for time periods not controlling for migration**

**Men**	Annual change (in months) in absolute gap in life expectancy between the most deprived 20% of local authorities and the rest of England **[95%CI]**		
During (2004-2012)	-1.03	[-1.45,-0.61]	<0.001
After (2013-2014)	0.25	[-1.21,1.72]	0.73
N=3564 LA years, R^2 0.67			
**Women**			
During (2004-2012)	-0.66	[-1.10,-0.21]	<0.001
After (2013-2014)	0.05	[-0.98,1.09]	0.92
N=3564 LA years, R^2 0.58			

B. Estimates for time periods controlling for migration

**Men**	Annual change (in months) in absolute gap in life expectancy between the most deprived 20% of local authorities and the rest of England **[95%CI]**		
During (2004-2012)	-1.06	[-1.50,-0.63]	<0.001
After (2013-2014)	0.29	[-1.14,1.73]	0.69
N=3564 LA years, R2 0.67			
**Women**			
During (2004-2012)	-0.66	[-1.11,-0.21]	0
After (2013-2014)	0.07	[-0.97,1.11]	0.89
N=3564 LA years, R2 0.58			

Appendix 5. Simulation study investigating likely errors that would result from using a comparison between Spearhead and non-Spearhead areas to identify a change in the trend in spatial socioeconomic inequalities.

We compared life expectancy in the most deprived quintile of local authorities to the rest of the country, between 1983 and 2015, to investigate trends in geographical health inequalities before, during and after the health inequalities strategy. We outline below the reasons why we used this grouping based on income deprivation rather than comparing the Spearhead areas to the rest of the country and provide a simulation analysis to test our assumptions.

The Spearhead areas were selected as local authority areas that were in the bottom fifth nationally for three or more of the following five indicators, between 1995-1997:

- Male life expectancy at birth
- Female life expectancy at birth
- Cancer mortality rate in under 75s
- Cardio Vascular Disease mortality rate in under 75s
- Index of Multiple Deprivation 2004 (Local Authority Summary), average score

These LAs were therefore not just identified because they were socioeconomically deprived but also because they were outliers for low life expectancy and high premature mortality in 1995-1997. There are a number of reasons why the change in the gap in life expectancy between Spearhead areas and the rest of the country may not reflect trends in spatial socioeconomic inequalities (i.e the difference in health between areas defined purely by their socioeconomic conditions).

Firstly as Spearhead areas were selected in part because they had low life expectancy and high under 75 year old mortality in 1995-1997, falls in life expectancy in Spearhead areas during the strategy period may be due to 'regression to the mean' rather than the effect of the strategy. In other words there could be a significant narrowing of the gap between Spearhead areas and the rest of the county when there was no narrowing of socioeconomic inequalities. Secondly , because the selection criteria also mean that Spearhead LAs had a relatively wide range of levels of socioeconomic deprivation there could be no significant narrowing of the gap between Spearhead areas and the rest of the county whilst there was a significant narrowing of socioeconomic inequalities.

To test these two potential sources of bias we conducted two simulation analyses. Firstly we simulated 100 datasets which were the same as the study data except that there was no difference in the trend in male and female life expectancy between LAs between 1983 and 2015 – i.e there are parallel trends with random variation simulated based on the variance in life expectancy within LAs found in the data. We then simulated cancer and circulatory mortality rates based on the correlations found in the study data. For each iteration, we applied the Spearhead selection criteria – identifying the LAs that are in the bottom fifth nationally for three or more of the five indicators between 1995-1997, and then conducted the analysis using this 'Spearhead' grouping and separately using our preferred grouping based solely on the income deprivation score of the IMD2004. By design in these simulations the only difference in the trends between local authorities is due to random noise – there is no narrowing of socioeconomic inequalities.

In these simulations 73% of the Spearhead models reported a significant narrowing of the gap during the strategy period, although there was actually no difference in trends in life expectancy, by design. This reflects the effect of regression to the mean. Only 6% of the deprivation models reported a significant narrowing of inequalities in these simulations. This

is approximately what would be expected since we are using a 5% threshold to define statistical significance (see Table A10).

Secondly we the simulated a further 100 datasets where life expectancy for each LA in each year was drawn from a random normal distribution such that the gap in mean life expectancies between LAs was set to narrow between the most deprived areas and the rest of the country, during the strategy period (2003-2012). We then simulated premature cardiovascular and cancer mortality for 1995-1997 as above, applied the Spearhead selection criteria in each iteration and conducted the two analyses (1) comparing 'Spearhead' and 'non-Spearhead areas' and (2) comparing income deprived and non-income deprived areas.

In these simulations 26% of the Spearhead models failed to detect a narrowing of inequalities (when there was one by design), whilst none of the deprivation models failed to detect a significant narrowing of inequalities (see Table A10).

Differences in life expectancy between the most income deprived quintile of local authorities and the rest of the country provides a measure of geographical health inequalities, that would be sensitive to the impact of the strategy, whilst not being affected by the biases outlined above. Thirty-five of this group of 40 deprived local authorities (88%) were Spearhead areas. They will have therefore been affected by actions targeted at the Spearhead areas as well as other broader policies that were targeted more generally at more deprived groups and areas (e.g. The allocation of additional NHS and local government resources to deprived areas, Sure Start, Health Action Zones, New Deal for Communities, introduction of minimum wage and tax and benefit changes). In addition, assessing progress on health inequalities, based on differences in health between groups defined by their socioeconomic status (e.g income), rather than their baseline health status, is more consistent with current definitions of health inequalities used in Europe and expert guidance on their measurement.[30–32]

Table A11. Results of 100 simulations for each scenario, showing the % of models indicating a significant reduction in the gap during the strategy period (when there was no difference in trends between LAs by design) and % of models indicating NO significant reduction in the gap during the strategy period when inequalities were simulated to narrow between the most deprived areas and the rest of the country.

	**Results from 100 simulated datasets for each scenario**			
	**Spearhead models**		**Deprivation models**	
**Simulated Scenario:**	Significant reduction in gap during strategy period. ($p<0.05$)	No significant reduction during strategy period.	Significant reduction in gap during strategy period. ($p<0.05$)	No significant reduction during strategy period.
**(1) No difference in trends between LAs**	73%	27%	6%	94%
**(2) Inequalities narrow between the most deprived areas and the rest of the country, during the strategy period (2003-2012).**	74%	26%	100%	0%

Appendix 6. Trend in life expectancy in Spearhead and non-Spearhead areas
When investigating the trends in life expectancy between Spearhead and non-Spearhead areas it is worth noting that because of the way they were selected, several Spearhead LAs were not particularly income deprived and the less income deprived Spearhead LAs tended to have lower life expectancy in 1995-1997 for their level of deprivation (see Figure A1).

Figure A1. Correlation between 1995-1997 life expectancy and income deprivation (IMD2004) for local authorities – showing that several Spearhead local authorities were not particularly income deprived and the less income deprived Spearhead LAs tended to have lower life expectancy for their level of deprivation.

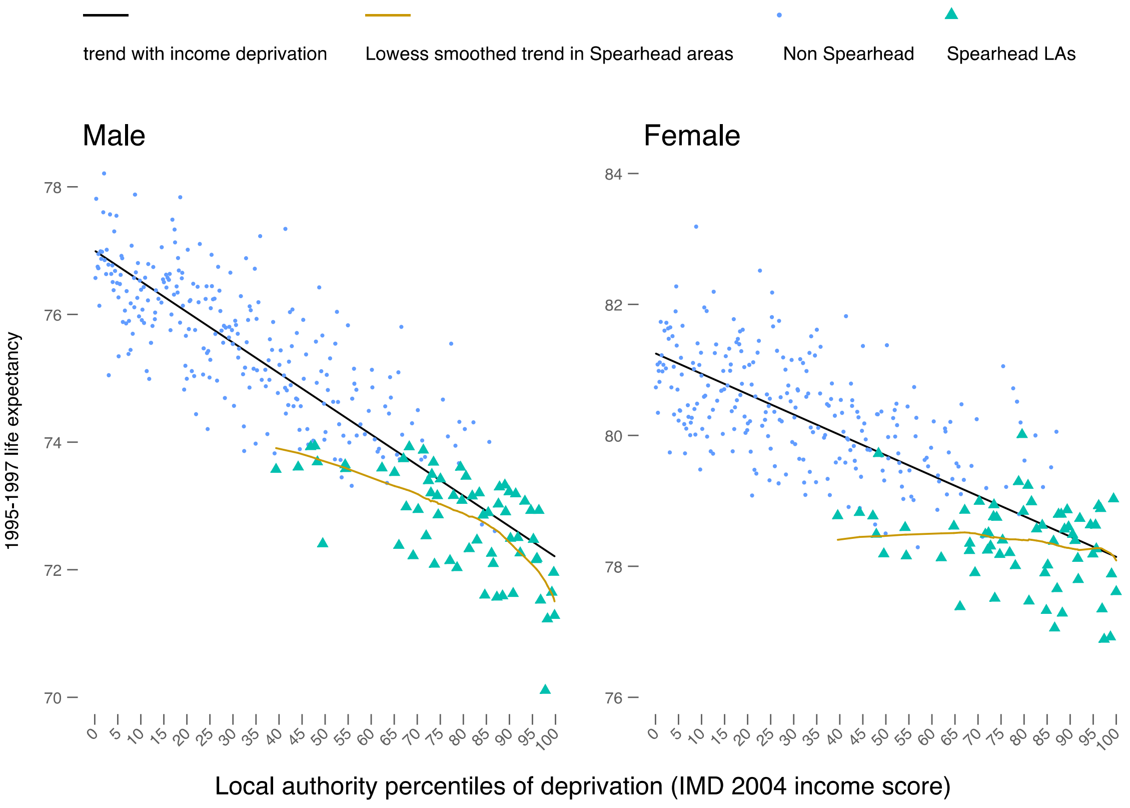

Figure A 2 shows that there was a change in the trend in the gap in male life expectancy between Spearhead and non-Spearhead areas during the strategy period, although this gap did not start to reduce until after 2006. The change in trend is less marked for female life expectancy. The target national target to reduce the gap in life expectancy at birth between the Spearhead Group of local authorities and the population as a whole (England), by at least 10% by 2010 (from a baseline of 1995-97), was achieved for male life expectancy – by 2009-2011. Table A 12 shows that the gap in male life expectancy was increasing significantly before 2004, it declined significantly during the strategy period and has increased since 2012. A similar pattern is seen for female life expectancy although the reduction in the gap during the strategy period was not statistically significant. Table A 12a

Figure A 2. Trends in life expectancy in the Spearhead local authorities and the rest of England as a whole and the relative and absolute differences 1983-2015.

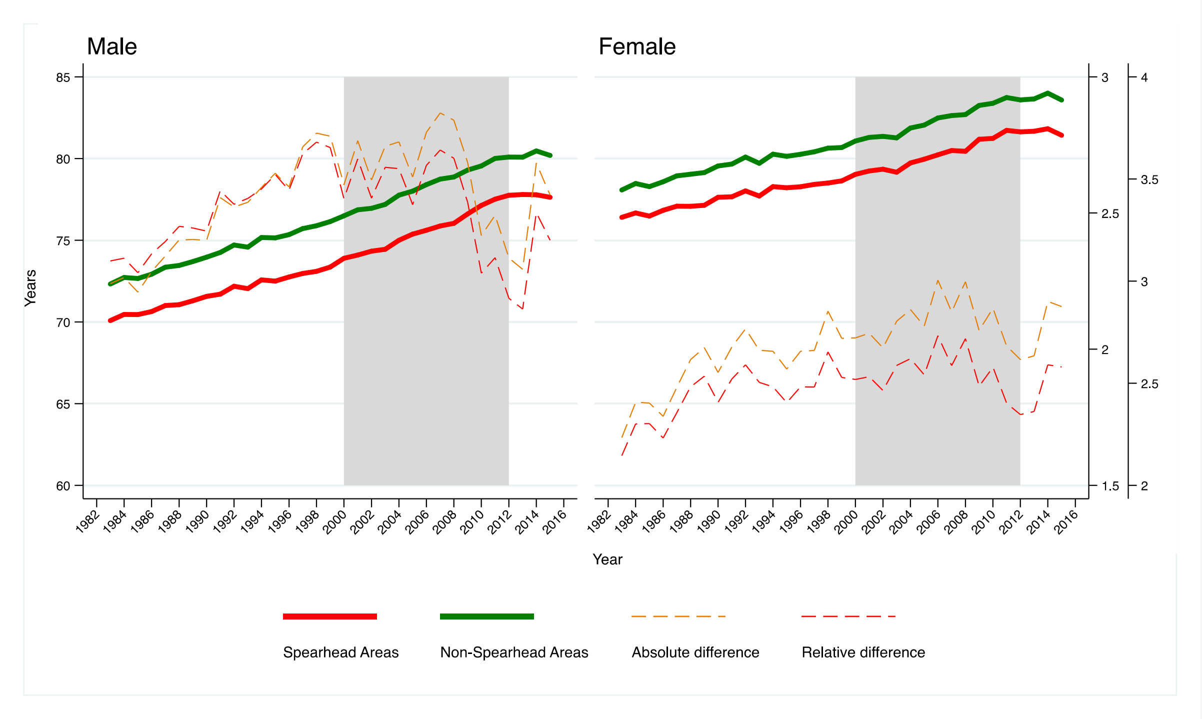

Table A 12. Trend in absolute inequalities in life expectancy, between the Spearhead area LAs and the rest of the country - before, during and after the health inequalities strategy. Trend is shown as the annual increase (+) or decrease (-) in the absolute gap in life expectancy measured in months.

**Men**	Annual change (in months) in absolute gap in life expectancy between the Spearhead Local Authorities and the rest of England **[95%CI]**		P-value for trend	P-value for change in trend from previous time period
Before (1983-2003)	0.43	[0.28,0.59]	<0.001	
During (2004-2012)	-0.52	[-0.78,-0.25]	<0.001	<0.001
After (2013-2015)	0.67	[-0.02,1.36]	0.06	<0.001
N=10692 LA years, R^2 =0.94				
**Women**				
Before (1983-2003)	0.19	[0.05,0.34]	0.01	
During (2004-2012)	-0.11	[-0.37,0.15]	0.42	0.09
After (2013-2015)	0.43	[-0.08,0.94]	0.1	0.08
N=10692 LA years, R^2 =0.89				

Table A 12a. Additional increase in life expectancy in Spearhead areas after controlling for the differential trends in deprived and non-deprived areas. Regression model as given in Appendix 2 with the addition of an interaction term between a dummy variable indicating Spearhead local authority and the period after 2005.

	**Additional increase in life expectancy in Spearhead areas relative to non-spearhead areas after 2005 (in months)**	95% CI	p-value
**Men**	2.75	[0.02,5.48]	0.05
**Women**	3.14	[0.97,5.31]	<0.001

Figure A3.

Appendix 7. Identifying 'natural' breakpoints in the trend in inequalities.

As the strategy developed incrementally and it is likely that there was a lag between implementation and any impacts on life expectancy, it was not possible to determine apriori precisely at which time points we might expect the trend in inequalities to change. We

therefore investigated empirically whether there was a significant change in the trend in health inequalities around the time of the beginning of the strategy period (between 1997 and 2006) and around the time of the end of the strategy (between 2008 and 2015). We use an iterative search procedure to identify which combination of two breakpoints – one at the beginning and one at the end of the strategy provided the best fit for the data by comparing all models with these alternative breakpoints, as well as models with just one of these, or no breakpoints. We then plotted the R-squared values from each of these models to identify the combination of breakpoints that provided the best fit with the data. In other words we fitted 88 separate models each with a different initial and final break points. Figure A4 shows the R-squared from each of these models – indicating that an initial breakpoint at 2003 and a final breakpoint at 2012 provides the best fitting model compared to all the other alternative break points.

Figure A 4 R-squared from 88 regression models with different breakpoints indicating the best fitting model has an initial breakpoint at 2003 and a final breakpoint at 2012. i.e the model allowing the trend to change at these points was a better fit than the alternative models.

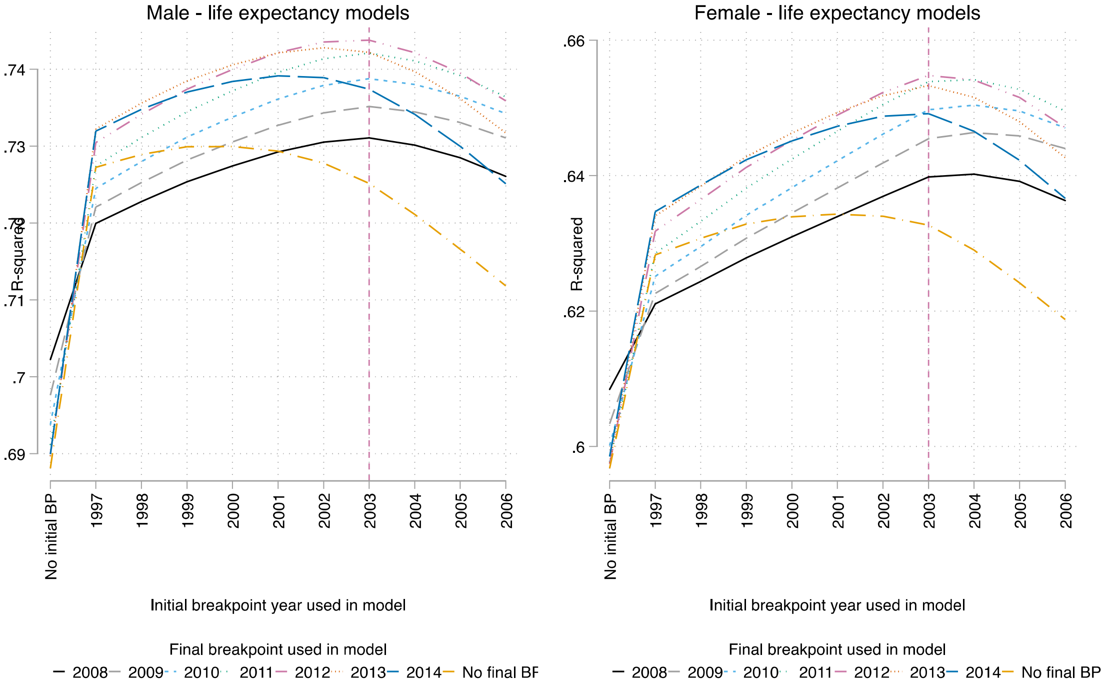

Investigating natural breakpoints within each of the two groups of local authorities.

To investigate whether there was a change in trend in either or both of the two groups of local authorities (1- deprived areas and 2- the rest of the country) before and after the strategy, we applied the same iterative search procedure separately for these two groups. Figure A 5 shows that there was a breakpoint for both groups of local authorities around 2003 and 2012. For trends in female life expectancy in deprived areas the R-squared is fractionally higher using a final breakpoint at 2013 rather than 2012 and for the trends in female life expectancy in non-deprived areas the R-squared is fractionally higher using an initial breakpoint of 2004 and a final breakpoint at 2011 rather than 2012, however these models are not a significantly better fit than models using breakpoints at 2003 and 2012.

This confirms what is shown in the full regression results in Appendix 4, that there was a significant upturn in the trend in life expectancy in both deprived and non-deprived areas around 2003. However this change in trend was greater in the more deprived areas – hence inequalities narrowed. Similarly there was a significant downturn in the trend in life

expectancy around 2012 across the country, but this change in trend was greatest in more deprived areas widening inequalities.

Figure A 5 R-squared from 176 regression models with different breakpoints for separate models (1) just including deprived local authorities and (2) just including the non-deprived local authorities in the rest of the country.

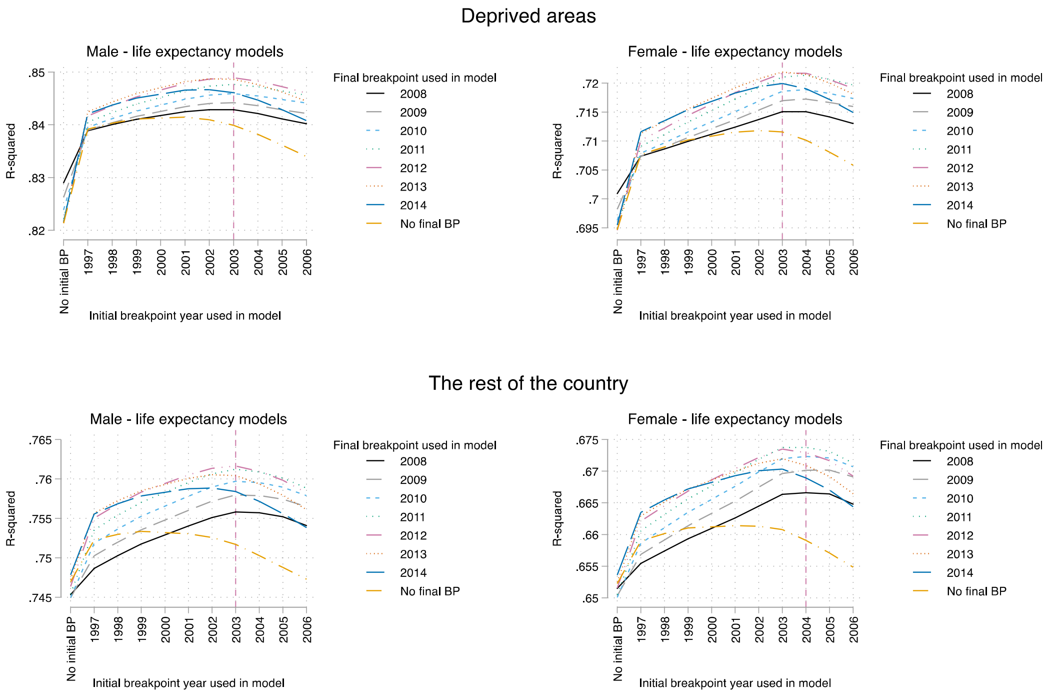

Appendix 8. Comparing the change in inequalities across neighbourhoods *within* the most deprived local authorities and the rest of England.

The decline in inequalities we observed between deprived and less deprived local authorities may not necessarily reflect a decline in inequalities at the neighbourhood or individual level. It is possible that inequalities between local authorities could be reduced if the health of more affluent groups within the deprived local authorities improved more than more deprived groups within those local authorities. In other words there could have been an increase in health inequalities within more deprived local authorities even though inequalities between local authorities reduced. To investigate whether this had occurred or not we analysed data on potential years of life lost (PYLL) in 1997-2001 and 2008-2012 for lower level super output areas (LSOA) obtained from the underlying indicators of the IMD2004 and the IMD2015. LSOA are small geographical areas used in reporting small areas statistics, each including a population of around 1600 people and 650 households. We only included LSOAs whose boundaries had not changed between the 2001 and 2011 censuses giving 31671 LSOA for analysis. We then calculated the change in PYLL for each LSOA between these two periods. We then plotted the change in PYLL against the IMD2004 income deprivation score for each LSOA, for England as a whole, for the most deprived local authorities and for the rest of England.

As Figure A6 shows there tended to be a greater decline in premature mortality in the more deprived neighbourhoods, reducing inequalities. Within the most deprived local authorities there was actually a greater decline in inequalities, than was observed in the less deprived local authorities. This suggests that the decline in inequalities observed at the local authority level following the English health inequalities strategy was also observed at the neighbourhood level and that this was achieved in part through reducing inequalities within

deprived local authorities as well as between these local authorities and the rest of the country.

Figure A 6 Change in PYLL by LSOA between 1997-2001 and 2008-2012 within most deprived local authorities and the rest of England.

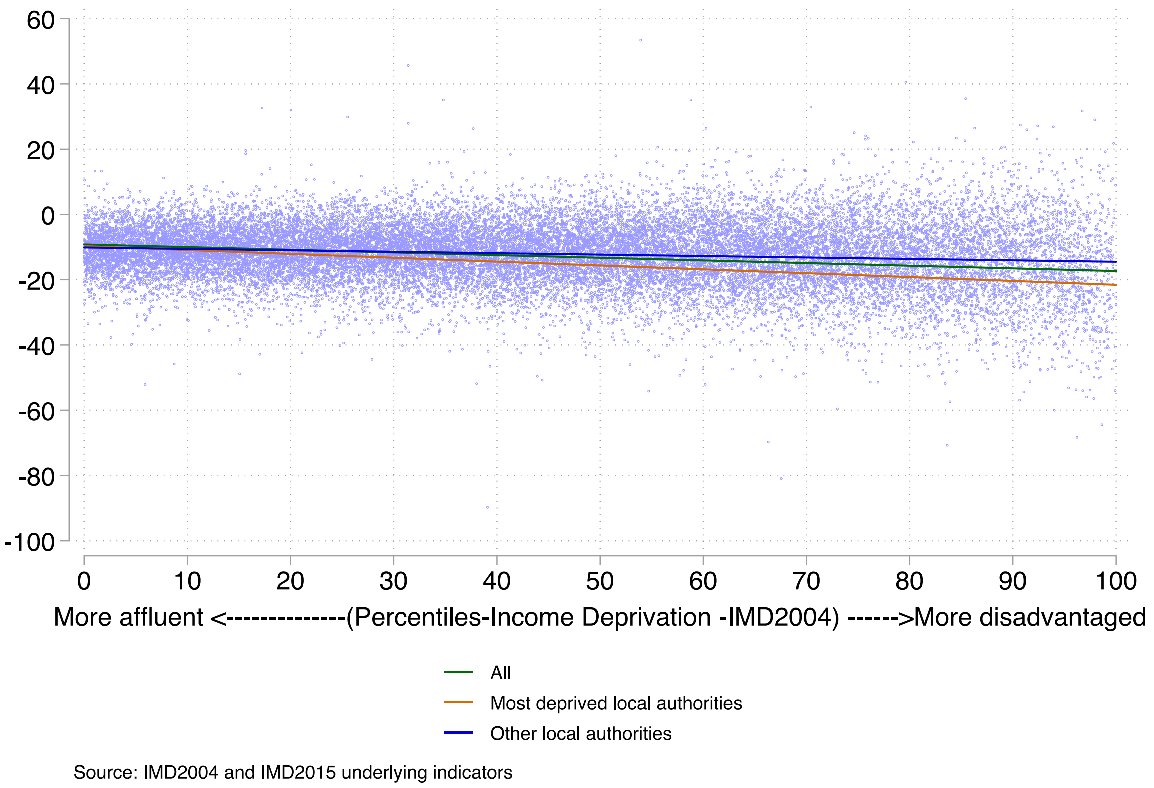

Appendix 9. Trend in inequalities, poverty measures and government expenditure.
Figure A7 shows that income inequalities as measured by the Gini index increased from 1979 to 1990. Although the Gini index remained stable from then on, poverty amongst pensions and children fell substantially from the mid-1990s to 2010. These reductions in poverty were the result of specific tax and benefit measures.[4] Total government expenditure increased markedly between 1997-2010 (see Figure A 8). This was particularly due to increases in spending on health and education, spending on housing and community amenities also increased markedly during this period (see Figure A 8). Part of the strategy was that the distribution of this increase in resources was equity-focused and targeted at the most deprived areas. As can be seen from Figures A8 and A10 increases in NHS and Local Government funding were particularly targeted at the most socio-economically deprived areas rather than at the Spearhead areas specifically. Both the increases in funding and the reductions in poverty could have contributed to the reductions in health inequalities that were observed in this study.

Figure A 7 Trend in unemployment and employment 1983 to 2015

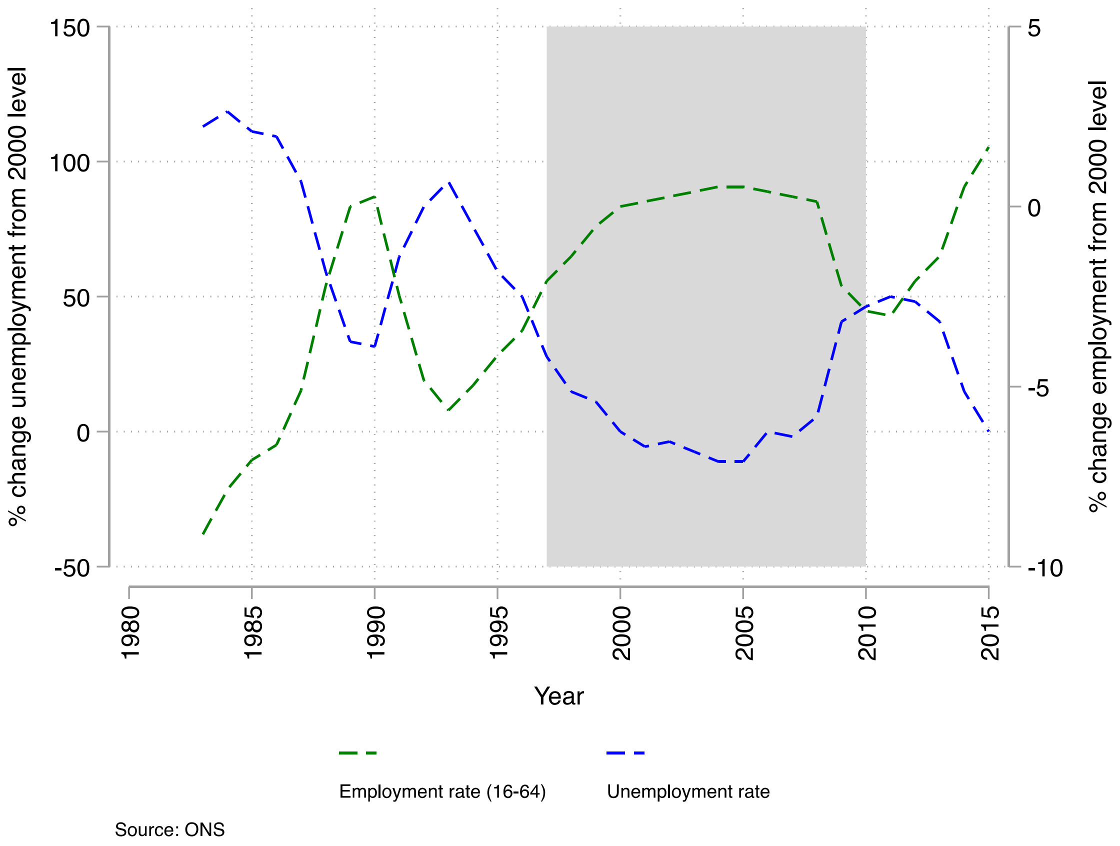

Figure A 8. Trend in inequality and poverty measures 1983 to 2014 (most recent year available).

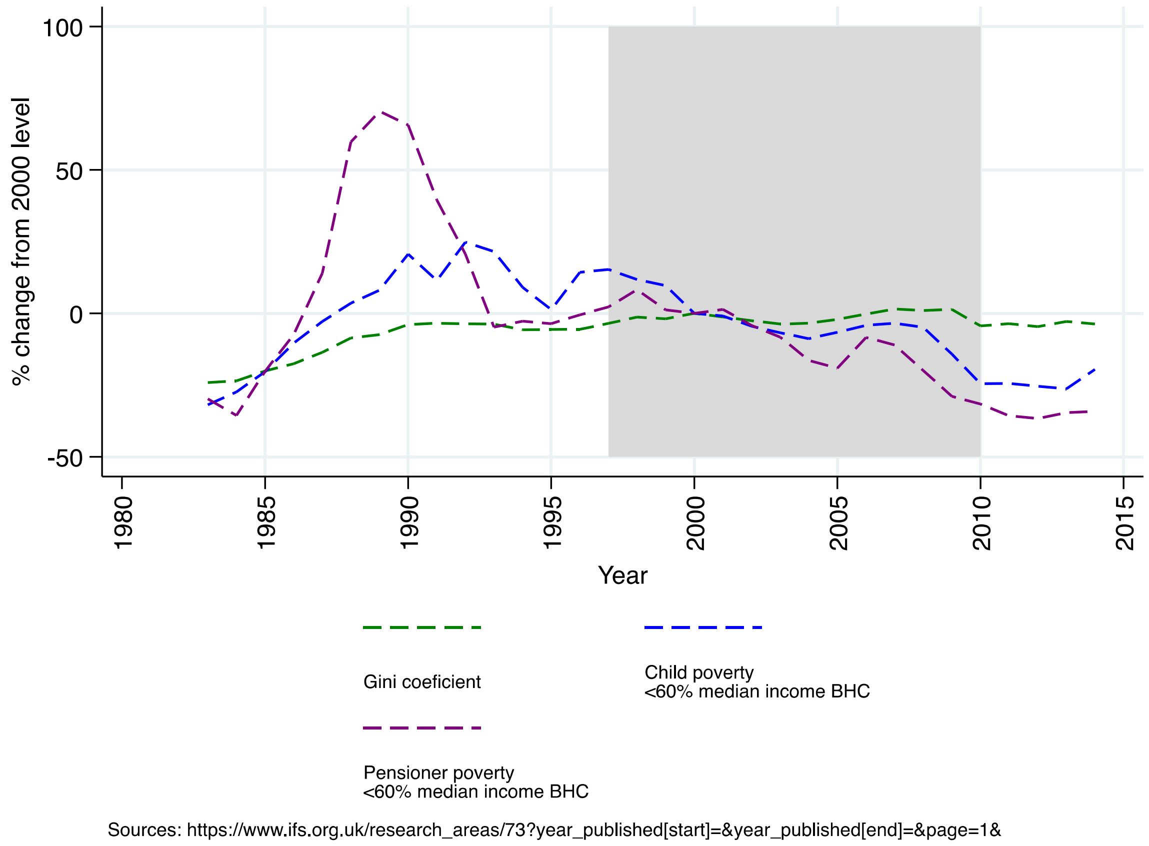

Figure A 9. Trend in public expenditure 1983 to 2015.

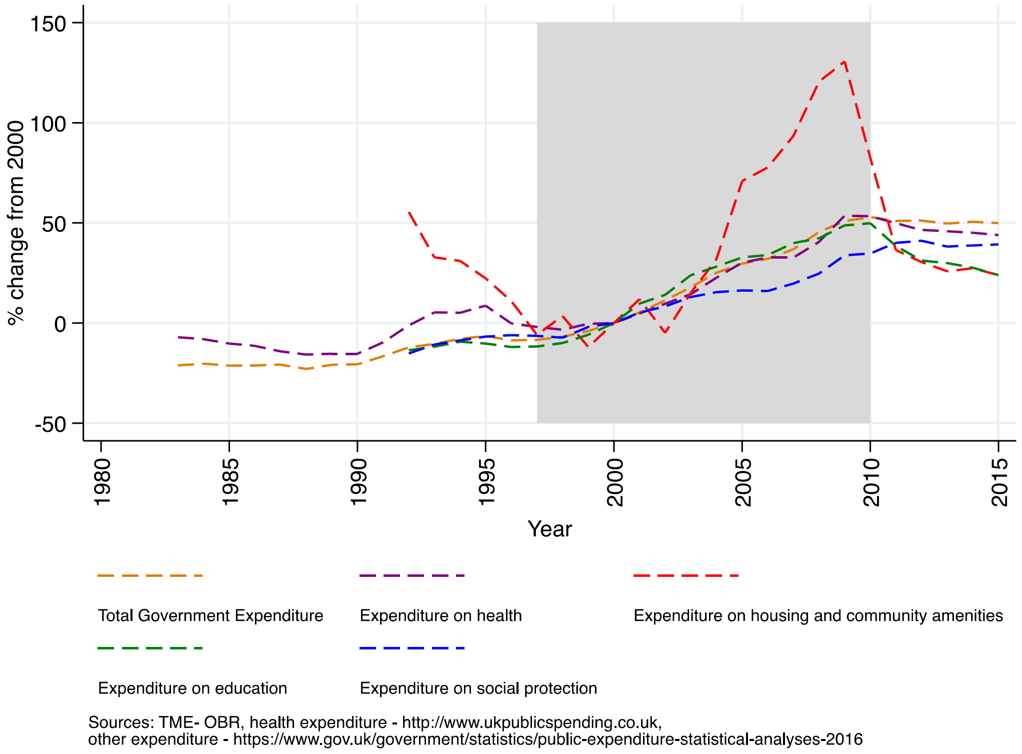

Figure A 10. Increase in NHS expenditure 2001-2010 for local authority areas (lower tier –districts and unitaries) by level of income deprivation. (Source: Authors own calculations using data compiled for Barr (2014)[5])

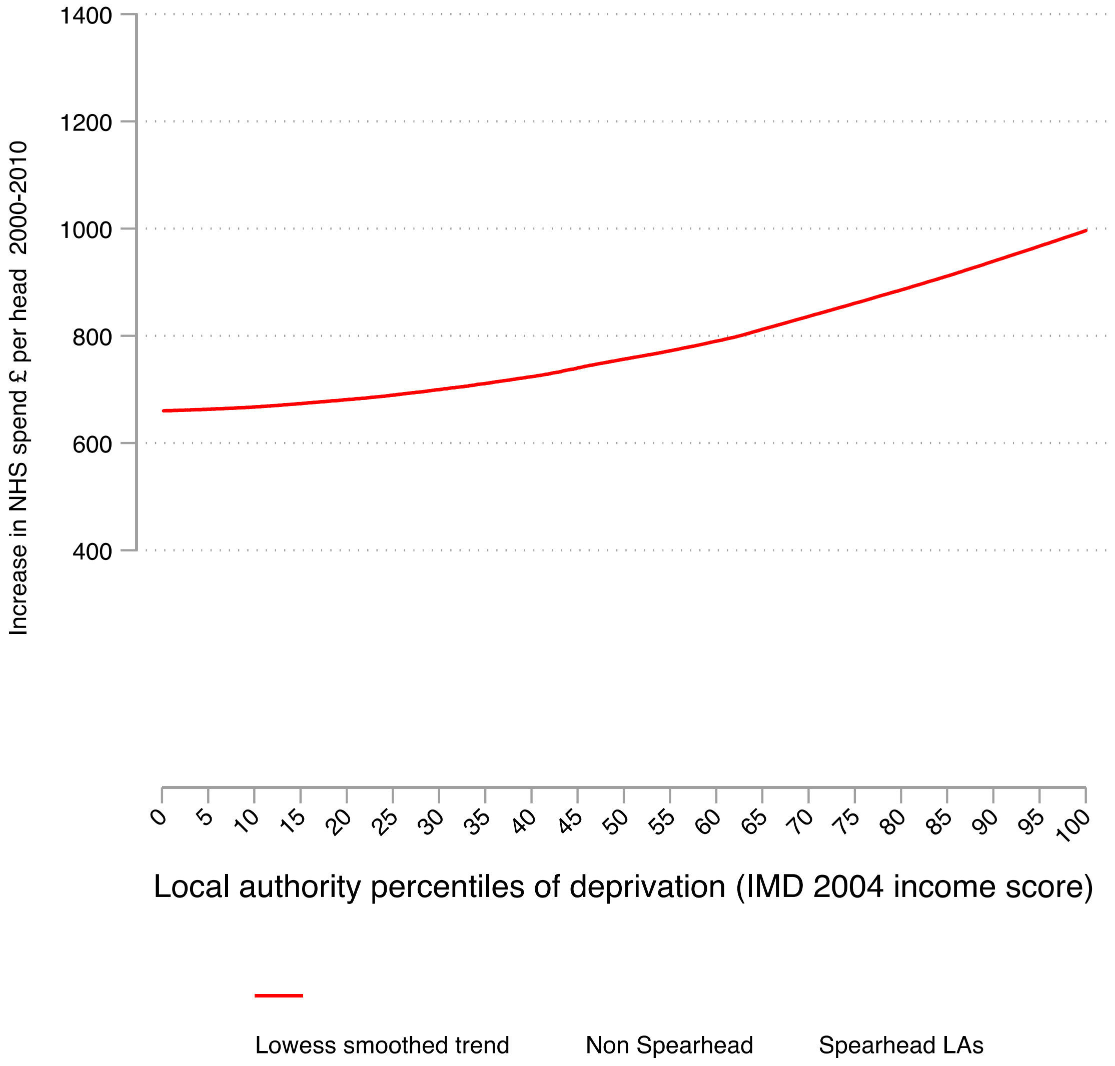

Increase in NHS spend £ per head 2000-2010
1400
1200
1000
800
600
400
0
5
10
15
20
25
30
35
40
45
50
55
60
65
70
75
80
85
90
95
100
Local authority percentiles of deprivation (IMD 2004 income score)
Lowess smoothed trend
Non Spearhead
Spearhead LAs

Figure A 11. Increase in local government (upper tier – counties and unitaries) expenditure 2002-2011 by local authority area level of income deprivation. (Source: Authors own calculations using data from the Department for Communities and Local Government.[6])

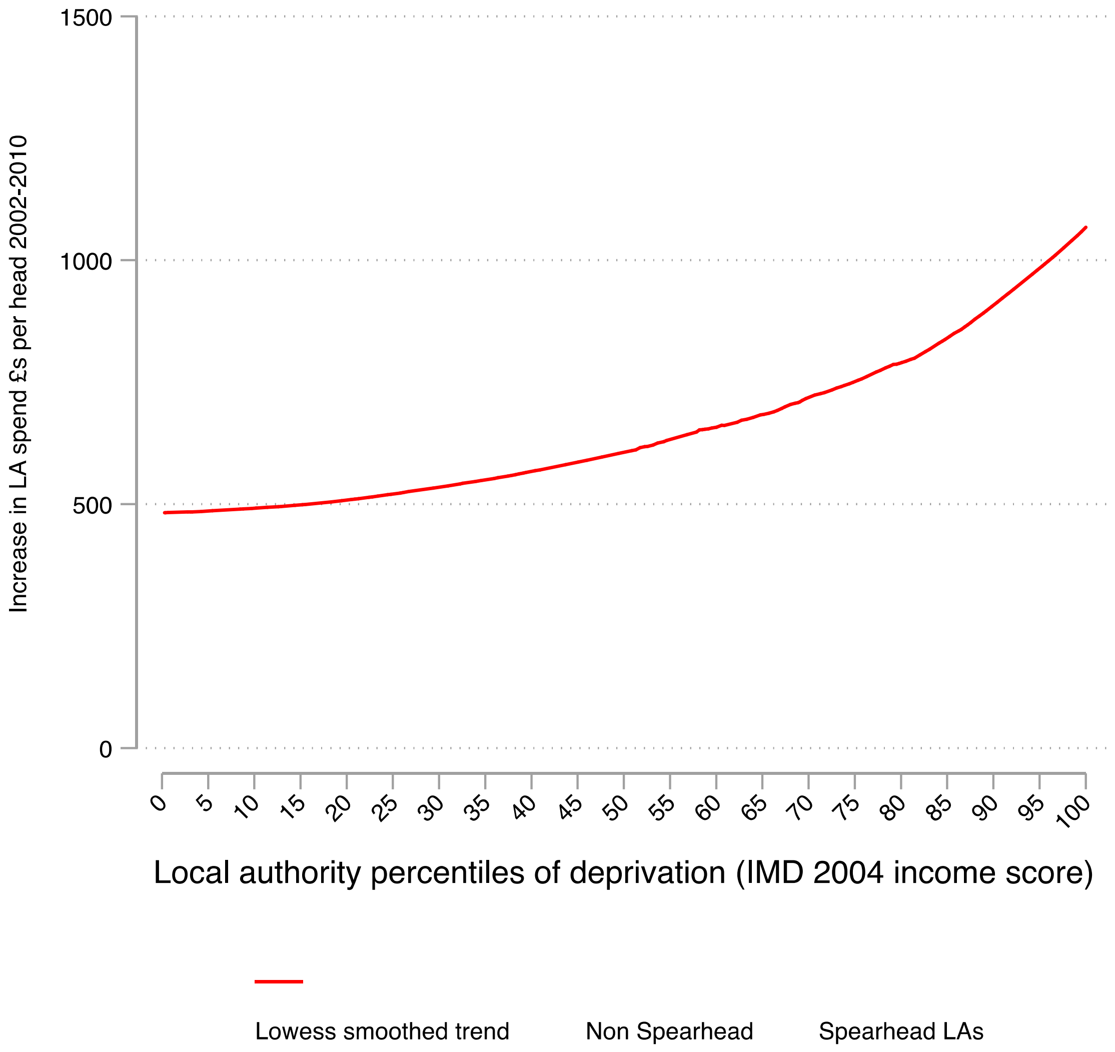

Appendix 10. Analysis showing the effect of population revisions on the gap in life expectancy between the most deprived LAs and the rest of England between 2006-2010.

To investigate the effect of population revisions following the 2011 census on the trend in health inequalities we recalculated life expectancies, for deprived areas and the rest of the country, using the old unrevised population estimates and compared the trend in the inequality gap using these estimates with the trend using the revised and more accurate population estimates. The gap was reduced slightly, from 2006 using the new population estimates (Figure A12).

Figure A 12. The gap in life expectancy between the most deprived LAs and the rest of England between 1990-2010, before and after population revisions following the 2011 census.

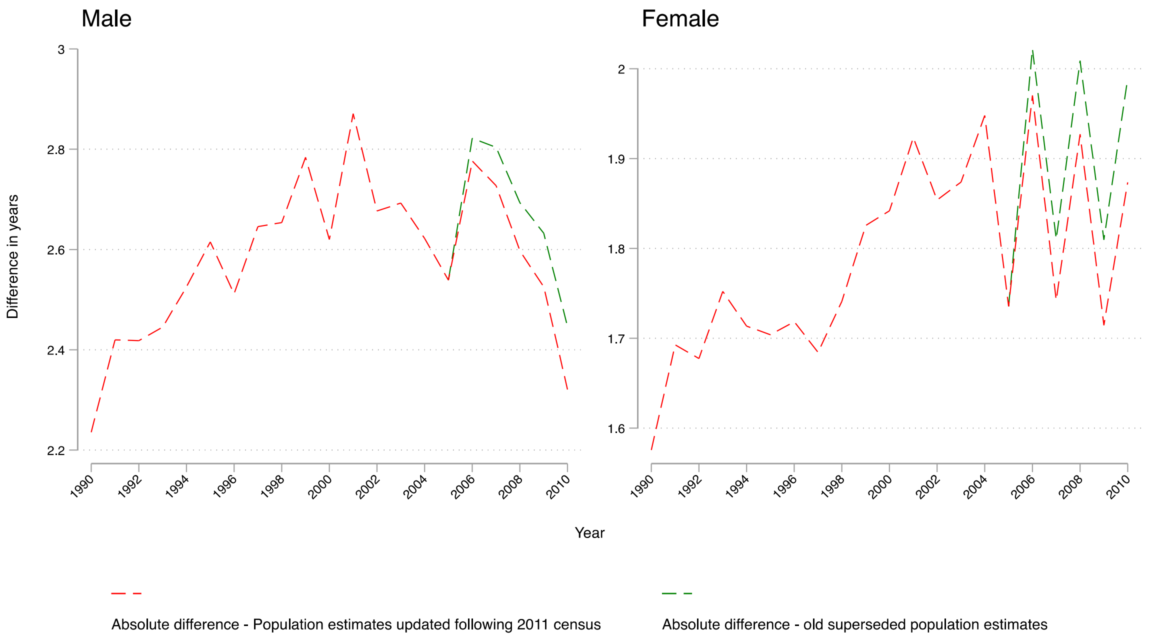

Appendix 11. Trend in life expectancy and descriptive data for the deprived group of local authorities and the rest of England.

Figure A 13. Trends in life expectancy in the most deprived local authorities and the rest of England and the relative and absolute differences 1983-2015. Male and female life expectancies on different scales to show detail of time trends.

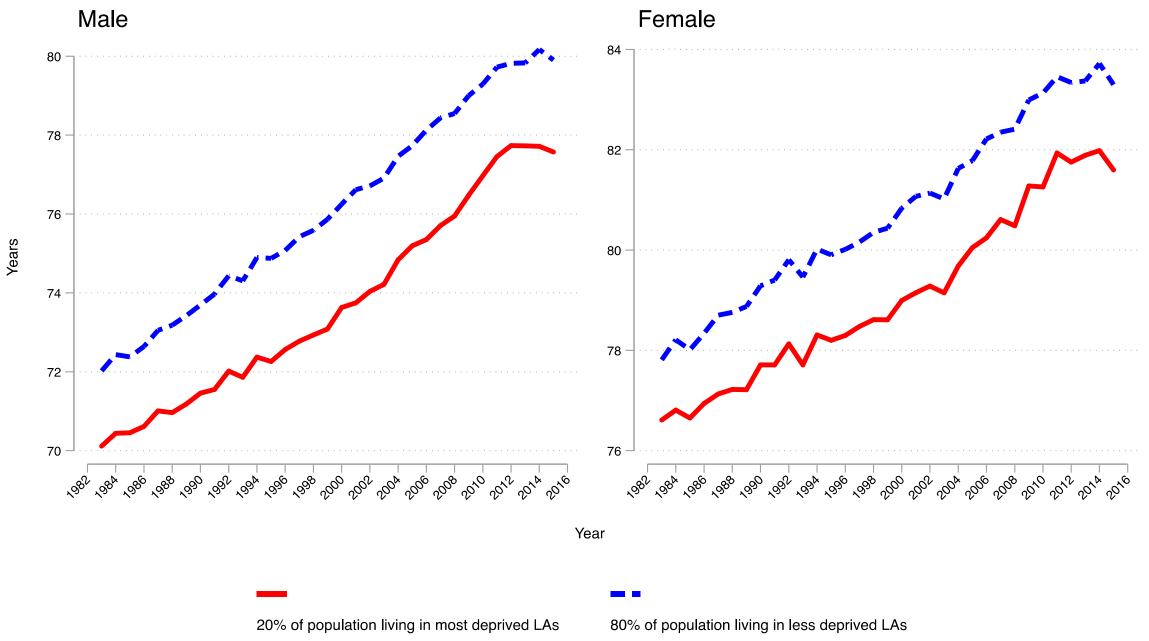

Table A 13 Descriptive statistics for the most deprived local authorities and the rest of England, 1983-2015

	**Deprived areas**	**The rest of England.**
**Mean annual population size (range)**	254504 (74,400-1,111,307)	138319 (29,305- 774060)
**% population living in urban areas.**	98%	71%
**Mean annual male life expectancy (range)**	73 (68-83)	76 (68-84)
**Mean annual female life expectancy (range)**	79 (74-87)	81 (74-88)
**Mean annual number of deaths (range)**	2599 (905-12775)	1430 (269-9089)

Figure A 14. Map showing the location of the most deprived local authorities , based on the IMD2004 income score, that included approximately 20% of the population of England (population weighted quintile).

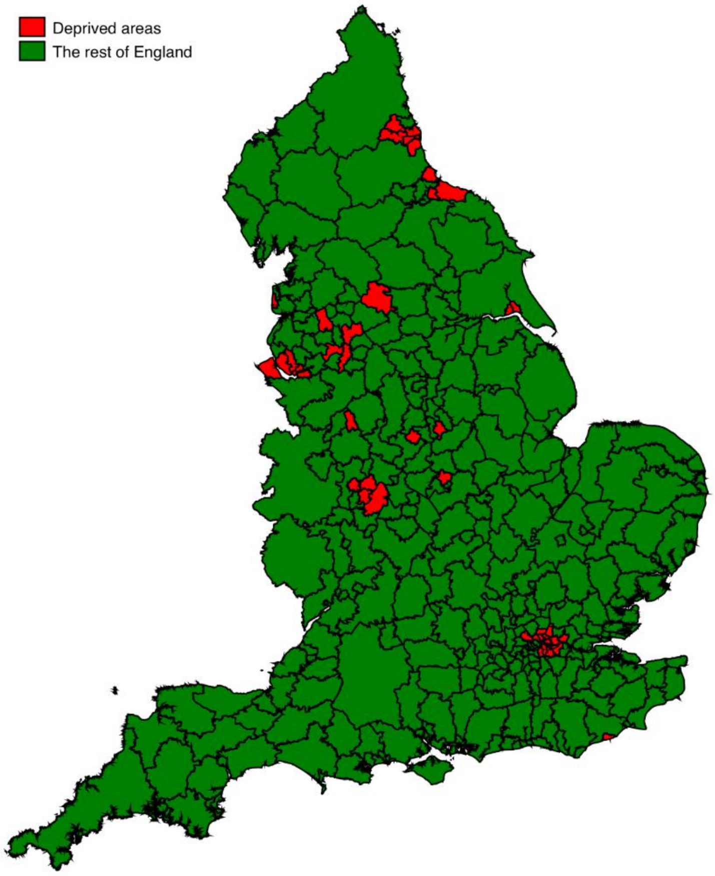

Appendix 12. Age specific trends in inequalities.

To investigate whether the trends in inequalities in life expectancy we observed were due to a change in inequalities in mortality in particular age groups we replicated our model using age adjusted mortality rates for 5 age groups 0-19 year olds, 20-44 year olds, 45-64 year olds, 65-74 year olds and over 75 year olds. We then added three way interaction terms to the model between age group, deprivation area, and time trend spline terms. We log transformed the age adjusted mortality rates in order to estimate the trend in relative inequalities in mortality rates, as relative measures are more comparable between age groups.

Figure A15 shows that the reduction in inequalities during the strategy period was particularly due to reduced inequalities in mortality in people under the age of 65. The reversal in this trend has largely been in the same age groups, although inequalities in female 0-19 year old mortality continued to decline.

Figure A 15- the trend in the relative difference in age specific mortality rates between the most deprived areas and the rest of the country before, during and after the strategy period, For 5 age groups (0-19 year olds, 20-44 year olds, 45-64 year olds, 65-74 year olds and over 75 year olds).

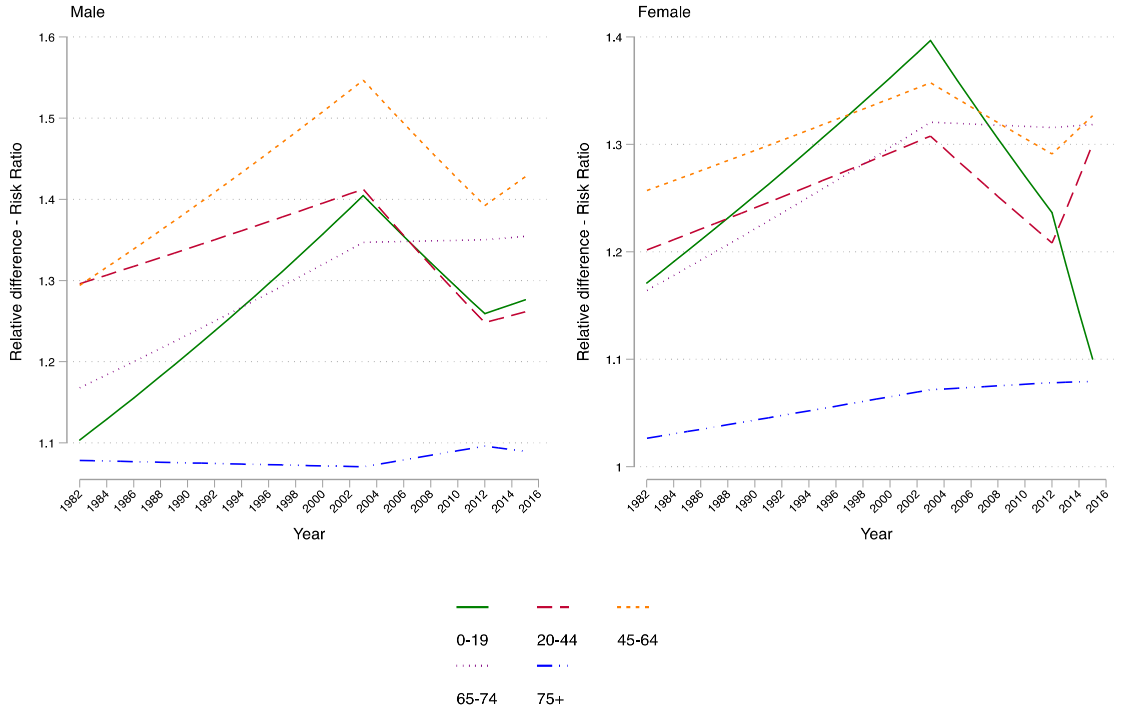

Appendix 13. Relative change in deaths under 65.
As a sensitivity analysis to check whether our results are influenced by changes in the population estimates over time rather than changes in the number of deaths, we replicated our model using the log of the number of deaths in each LA as the outcome. The model then provides an estimate of the annual change in the relative percentage gap in deaths under 65 between the most deprived 20% of local authorities and the rest of England before, during and after the health inequalities strategy. As this analysis does not use population denominators it cannot be influenced by inaccuracies in population estimates.

Table A 14. Annual change in the relative percentage gap in deaths under 65 between the most deprived 20% of local authorities and the rest of England before, during and after the health inequalities strategy. Trend is shown as the annual increase (+) or decrease (-) in the percentage difference in life expectancy between deprived LAs and the rest of the country.

**Men**	Annual change in the relative percentage gap in deaths under 65 between the most deprived 20% of local authorities and the rest of England **[95%CI]**		
Before (1983-2003)	0.545	[0.071,1.018]	0.024
During (2004-2012)	-0.757	[-1.297,-0.218]	0.006
After (2013-2015)	1.75	[0.203,3.298]	0.027
N=10692 LA years, R^2 =0.24			
**Women**			
Before (1983-2003)	0.228	[-0.169,0.625]	0.259
During (2004-2012)	-0.619	[-1.121,-0.118]	0.016
After (2013-2015)	1.668	[-0.123,3.458]	0.068
N=10692 LA years, R^2 =0.89			

Note: Estimates based on fixed effects regression model using a local authority panel dataset of life expectancy from 1983 to 2015.

Appendix 14. Investigating the non-linear relationship between deprivation and increase in life expectancy before, during and after the strategy

To explore how improvements in life expectancy varied across levels of deprivation during each period we used kernel-weighted local polynomial regression models to plot the average annual change in life expectancy, during the three time periods - before, during and after the strategy - against the population weight percentiles of deprivation for all local authorities.

Figure A16 shows the average annual change in life expectancy by percentiles of deprivation for the three periods. We can see that the relationship between improvements in life expectancy and deprivation prior to the strategy (1983-2003) were approximately linear – with life expectancy increasing at a faster rate in less deprived areas – increasing inequalities. During the strategy period (2004-2012) there was a non-linear relationship between improvements in life expectancy and deprivation with the greatest improvements in the most deprived areas. This is particularly true of the increases in female life expectancy. Recent increases in inequalities since 2012 have been experienced across the social gradient.

Figure A 16. Average annual change in life expectancy by percentiles of deprivation for three periods (1) 1983-2003, (2) 2004-2012 and (3) 2013-2015.

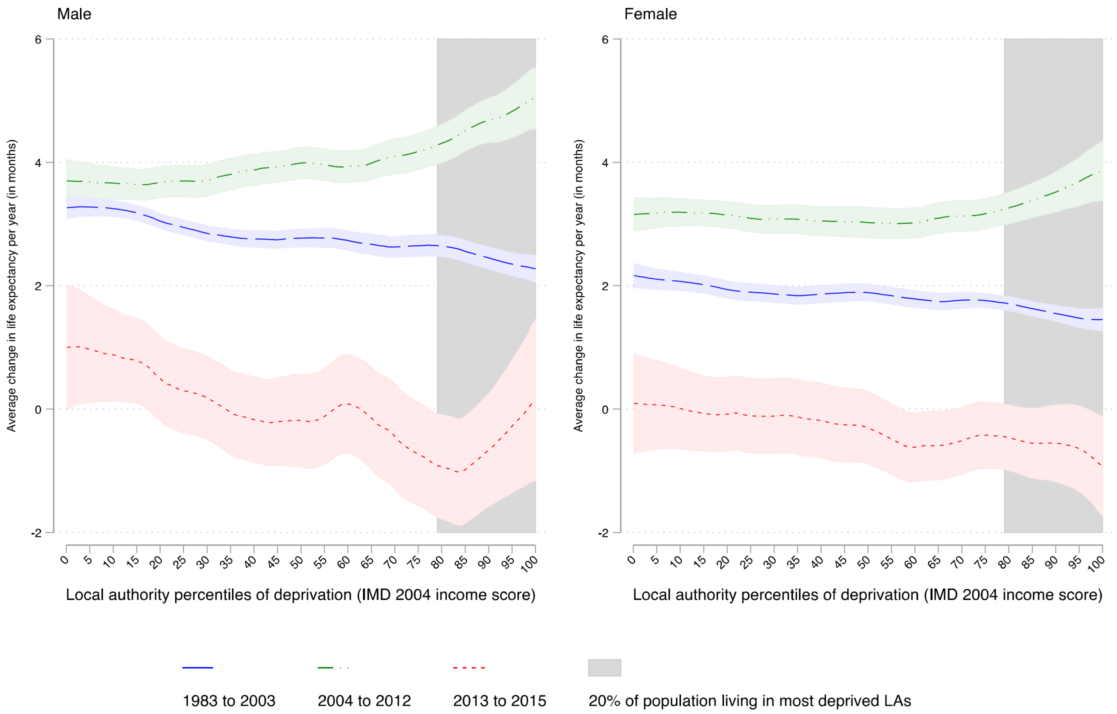

Supplement: Supplementary file 1 — Supplementary information: appendices 1-14 [file barb037192.ww1.pdf]
